# Self‐Assembly Enabled Mie‐Scattering in Hematite Nanoplatelet Stacks

**DOI:** 10.1002/nap2.70253

**Published:** 2026-08-13

**Authors:** Péter Márton, Dániel Zámbó, András Deák

**Affiliations:** ^1^ Photonics Laboratory HUN‐REN Centre for Energy Research Budapest Hungary; ^2^ Department of Physical Chemistry and Materials Science, Faculty of Chemical Technology and Biotechnology Budapest University of Technology and Economics Budapest Hungary

**Keywords:** enhancement, hematite nanoparticle, mie‐scattering, self‐assembly

## Abstract

In this work spectral changes following the self‐assembly of hematite nanoplatelets into particle stacks are characterized and interpreted. The individual hexagonal nanoplatelets are dispersed in aqueous medium and have a dimension of 120 × 20 nm. In the framework of Mie‐scattering, hematite is considered as a medium refractive index material in the visible wavelength range and the particles' ensemble optical spectrum is dominated by absorption with a small contribution from the electric dipole scattering. However, by introducing an increasing amount of inert electrolyte into the system, these platelets can be assembled into vertical stacks, dictated by the interaction‐area proportional attractive dispersion force. The process is accompanied by a profound change of the optical spectrum and the evolution of a new scattering peak is observed. Based on the simulation‐derived spectra and optical near‐field properties, this can be attributed to the stacking‐associated enhancement of the collective electric and magnetic dipole modes. This is confirmed by performing correlative electron microscopy and scattering spectroscopy on individual nanoparticles and particle stacks. The presented approach provides a yet unexplored strategy for modulating especially the magnetic mode of otherwise weak Mie‐scatterer nanoparticles.

## Graphical abstract

The spectral evolution during the electrolyte‐induced self‐assembly of 120 × 20 nm hematite nanoplatelets into vertical stacks in aqueous solutions is investigated. Optical simulations and correlative electron microscopy/single particle spectroscopy confirm that a collective magnetic dipole mode develops in the assembling stacks.

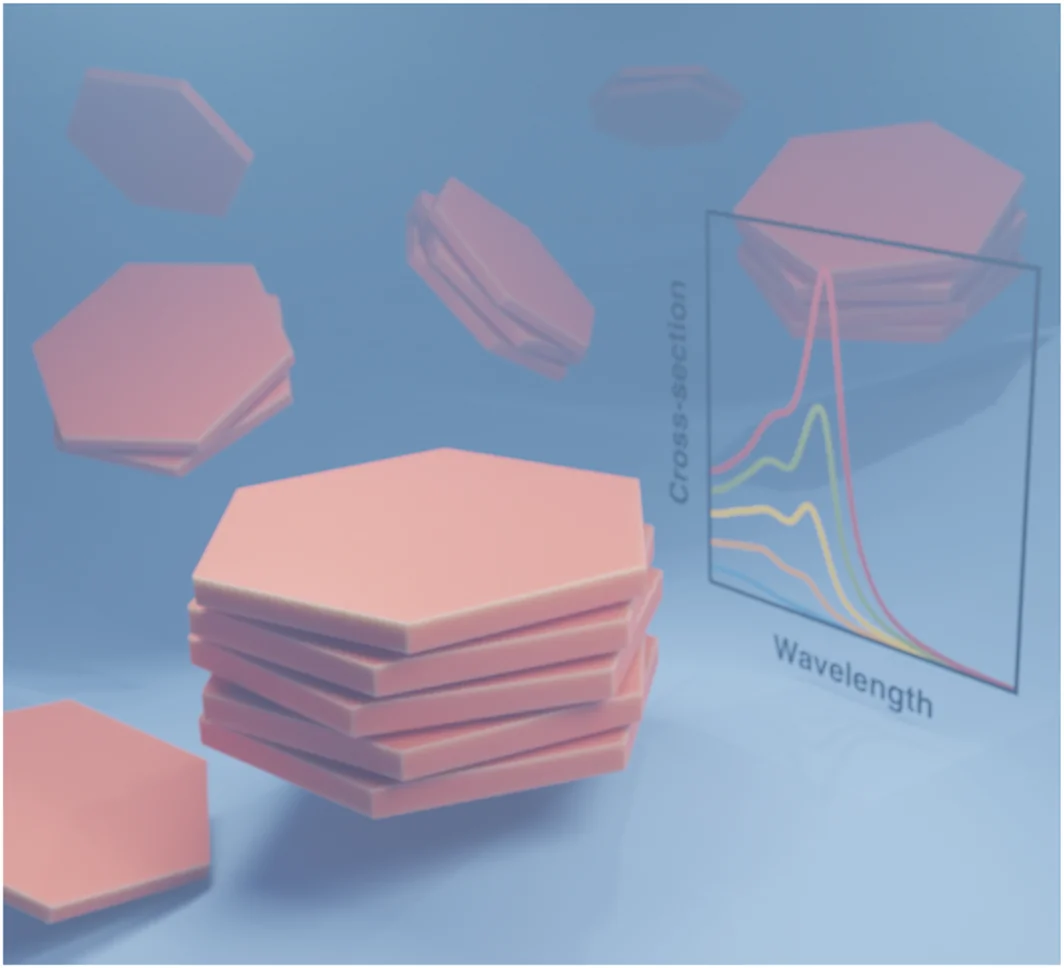

## 1 Introduction

Hematite is regarded as a material with great potential for various photoelectrochemical applications, as it is an easy to process, cheap, stable and earth abundant n‐type oxide semiconductor [1]. It is one of the top candidates for solar water splitting, where it is used as a photoanode [2]. Nevertheless, it suffers from low charge carrier mobility due to the formation of self‐trapped excitons [3], a poor minority (hole) diffusion length of about 2–4 nm [4], and a limited carrier extraction depth of approximately 10 nm [5]. Additionally, despite its appealing band‐gap (2.2 eV) and its alignment, the formation of polarons upon optical excitation limits the effective electronic band gap to ca. 1.8 eV, leading to lower‐than‐expected photovoltages and hence lower efficiencies [6]. Besides the photoanode use of hematite, utilization of its optical properties mainly focuses on optoelectronic application, such as solar‐fuel generation or catalysis [7, 8].

In addition to the optoelectronic application of hematite nanostructures, the material also gained attention as a candidate for nanoscale light modulation, owing to its mid‐range refractive index (around 3), rendering it a suitable Mie‐scatterer material for the visible wavelength range [9]. Engineered Mie‐scattering arising from dielectric materials has recently emerged as a rich field in photonics, allowing the access to a range of special optical nanoscale phenomena [10]. Regarding the photonic application of hematite, it has significant absorption around 400 and 550 nm, related to the transitions between the (hybridized) orbitals of O and Fe [11]. However, starting from 600 nm, absorption is very low and the complex refractive index is dominated by its real value; hence, low‐loss manipulation of light starting from around this wavelength becomes possible. The Mie‐scattering properties of spherical hematite nanoparticles were already studied both experimentally and using analytical formulae in the mid‐1980s [12]. It was pointed out already at that time that the size increase of spherical particles shifts the extinction peak maximum associated with Mie‐scattering from ca. 400–550 nm as the size of the spheres increases from 100 to 160 nm. Since then, with the development of synthesis procedures and more efficient simulation tools, a variety of hematite nanoparticle shapes have been prepared (e.g., rods, cubes, disks, etc.) and their properties have been investigated in detail [13, 14, 15, 16]. The general renewed interest in such dielectric nanoparticles is mainly due to the fact that such nanostructures—in contrast to their plasmonic counterparts—can also enable internal field localization and can be designed to overcome the inherent losses associated with metal supported localized plasmon resonances.

In this work we create vertically stacked hematite nanostructures by modulating the colloidal interaction between individual hematite nanoplatelets. It is shown that stacking of the particles results in pronounced changes in the optical spectra; a scattering peak associated with the collective magnetic dipole mode is developing in the aggregates, which is associated with the specific stacked arrangement of the platelets. The assembly enabled enhancement of the dipole modes is supported by optical simulations and the scattering increase confirmed by correlative electron and scattering optical microscopy investigation of individual particles and particle stacks.

## 2 Results and Discussion

The prepared nanoplatelets show a monomodal size distribution (Figure 1b) and have an average lateral size of 195 ± 20 nm. Their thickness is around 20 nm as measured on occasionally vertically oriented platelets. XRD measurements confirm the particles to be of hematite phase (Supporting Information S1: Figure S1).

**FIGURE 1:**
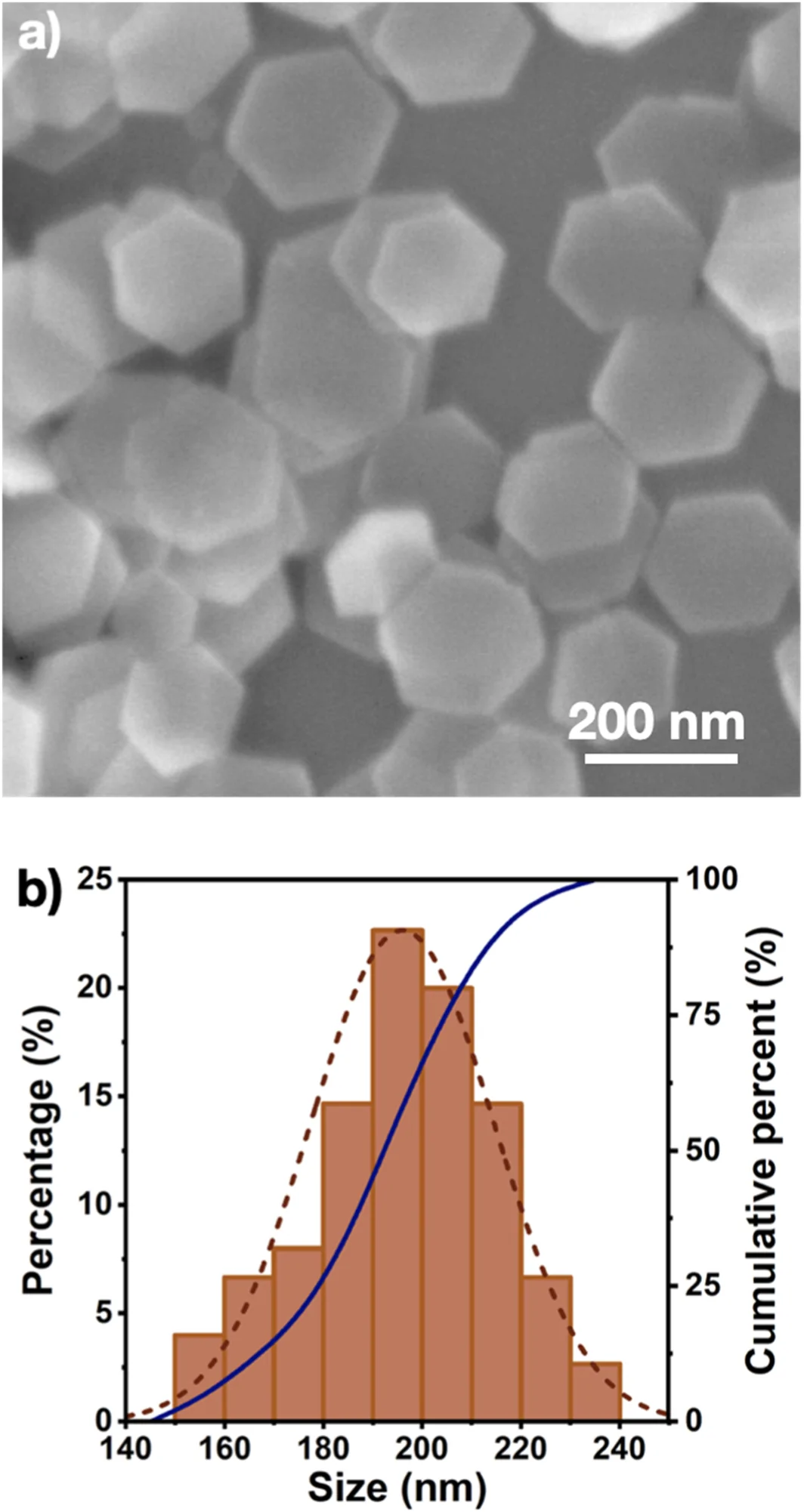
Scanning electron microscopy image of the prepared nanoplateles (a) and the distribution of the lateral size (tip‐to‐tip) (b).

The nanoplatelets are stable as indicated by the DLS derived time‐dependent size distribution and also no change in their ensemble optical spectra over time can be observed (Figure 2a,b). The spectra display two prominent features: a broader peak at around 450 nm and a shoulder at 575 nm. According to the simulations, the spectrum of a single platelet is dominated by absorption, especially at shorter wavelengths. Both the absorption and scattering cross sections have a maximum near 450 nm, resulting in a dominant peak at this wavelength (Figure 2c). Around the shoulder at 575 nm the absorption and scattering cross‐sections become comparable. The modulation of both cross‐sections with wavelength can be primarily ascribed to the variation in the dielectric function of hematite (Supporting Information S1: Figure S2). Looking at the contributions from the different modes to the scattering it becomes clear that the electric dipole (ED) mode dominates the scattering from the platelets, whereas the magnetic dipole (MD) and higher order modes (electric quadrupole—EQ, magnetic quadrupole—MQ) are minor or negligible in the investigated wavelength range (Figure 2d). Hence, the spectral features observed at around 300, 400, 450, and 575 nm are related to the interplay of the dielectric function and the increased cross‐section at these wavelengths: at 300 nm there is an increased imaginary component of hematite with the simultaneous presence of the quadrupole mode, whereas at around 400–450 and 575 nm the peaks originate from the simultaneous presence of dipole modes and the two prominent optical transitions in hematite (from O 2p and Fe t_2g_ to empty Fe t_2g_* and from Fe 3d e_g_ hybridized with O 2p to unoccupied Fe t_2g_* for the latter) [11].

**FIGURE 2:**
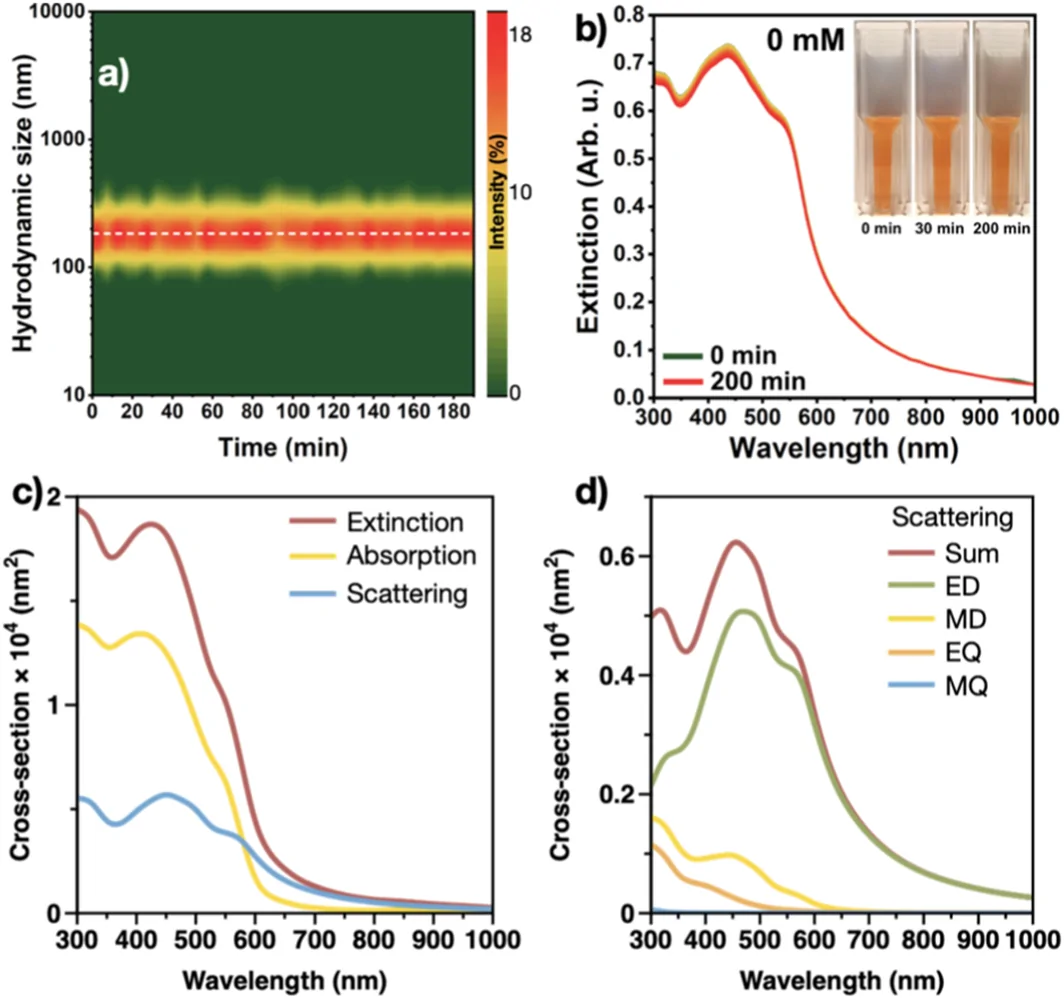
Time‐dependent dynamic light scattering derived size distribution (a) and ensemble optical spectra (b) of the as‐prepared particles. The dashed line in the DLS plot indicates the pristine nanoplatelet size. Simulated scattering, absorption and extinction cross‐section of a single nanoplatelet suspended in water (c) and contribution of the different modes (E, electric; M, magnetic; D, dipole; Q, quadrupole) to the scattering cross‐section (d).

The electrophoretic mobility of the as‐prepared particles was measured to be +2.21 μmcmV^−1^s^−1^, indicating that the kinetic stability of the hematite nanoparticle system is predominantly determined by a charge‐stabilization mechanism, which is in accordance with earlier results obtained for sphere‐like nanoparticles [17]. The charge stabilization is further supported by the fact that upon gradually increasing the inert electrolyte (KNO_3_) concentration of the system to 1 mM, the electrophoretic mobility gradually decreases to +1.68 μmcmV^−1^s^−1^ (Supporting Information S1: Figure S3). Interestingly, at 1 mM KNO_3_ concentration, initial signs of particle aggregation appeared, as indicated by a slight increase of the DLS‐derived average particle size from 183 to 250 nm (Figure 3a). However, the optical spectrum shows pronounced changes over time (Figure 3b). The most important feature change is that the shoulder in the spectrum around 575 nm gradually evolves into an extinction peak, and its intensity is becoming comparable with the originally dominant peak located at around 450 nm (the overall intensity drop with time is due to slow sedimentation). At 100 mM KNO_3_ concentration the size increase is clearly faster and the hydrodynamic radius increases to ca. 1 μm. This faster aggregation process also translates to a rapid change of the extinction spectrum, whereby the peak evolving around 575 nm gets even more pronounced. The scaling of the aggregation dynamics with the salt concentration is obvious as at 100 mM salt concentration there is significant aggregation based on DLS (Figure 3c) and while the characteristics of spectral changes are similar, it is also clear that the sample starts to sediment in the investigated time window (Figure 3d). At intermediate (5 and 10 mM) KNO_3_ concentrations both DLS and spectroscopic data are similar to the 100 mM case (Supporting Information S1: Figure S4), indicating the central role of the electric double layer stabilization and a kinetically determined aggregation process. Interestingly, stronger particle aggregation leads to a more pronounced peak around 575 nm, although its position remains nearly constant across all systems. There is, however, a slight shift of the long wavelength peak position on the order of a few nanometres, which matches the trend of the DLS‐derived size change (Supporting Information S1: Figure S5). These already indicate that the dominant optical interaction responsible for the observed change in the relative peak intensities might be the appearance of an aggregation induced collective optical mode.

**FIGURE 3:**
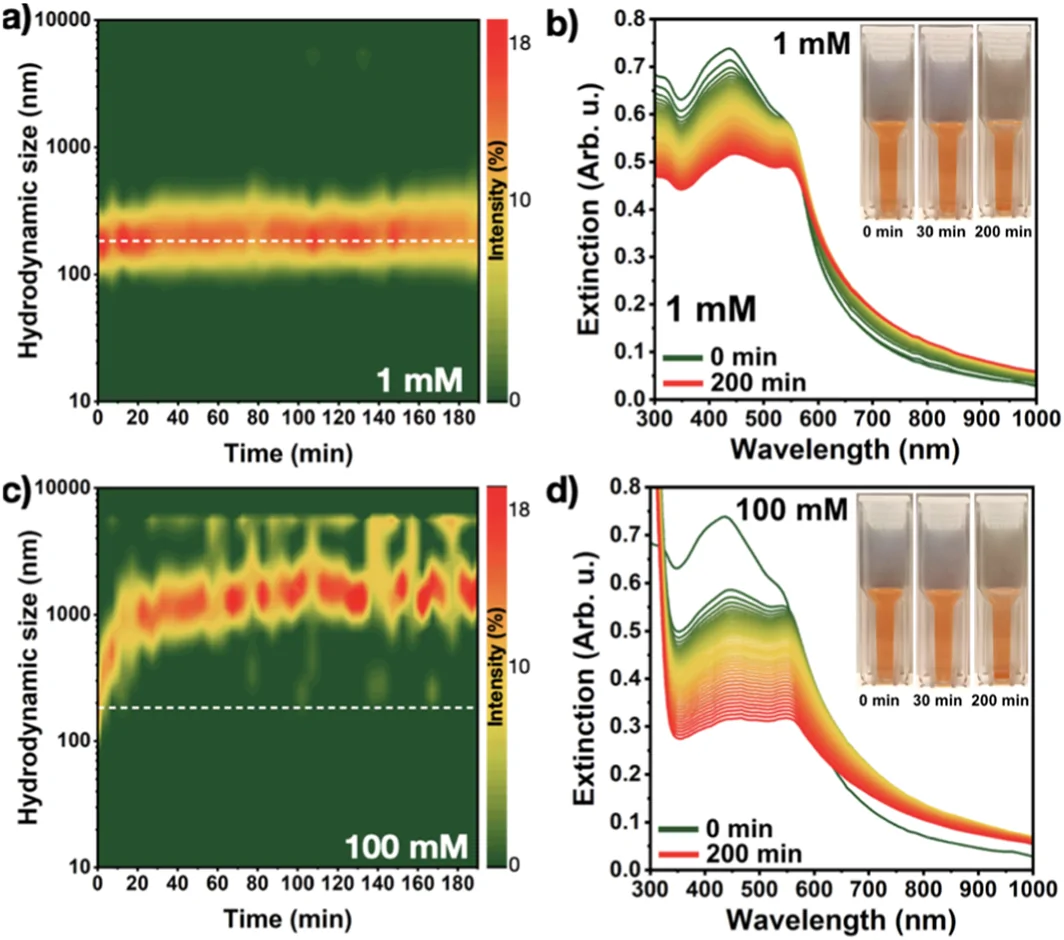
Time‐dependent dynamic light scattering derived size distribution (left) and ensemble optical spectra (right) of the particles recorded at 1 mM (a, b) and 100 mM (c, d) KNO_3_ concentrations. The dashed line in the DLS plots indicates the pristine nanoplatelet size.

To confirm that the observed spectral change is connected to formation of particle aggregates, scanning electron microscopy was employed on the aggregated systems. As shown in Figure 4, nanoplatelet stacks can be observed in the aggregated systems. Instead of a random particle network formation, well‐aligned structures are formed, which is consistent with the concept of interaction‐area proportional interparticle forces. As the system is destabilized by a decrease in the electric double layer repulsion, the net interparticle force becomes attractive due to the unchanged and ever‐present dispersion interactions. The minimum energy corresponds to an arrangement in which the geometrical overlap between the particles is maximized; consequently, face‐to‐face stacking of the platelets takes place, which is in stark contrast to the case of spherical particles, for which the directional uniformity of the interaction profile can lead to fractal‐like aggregates in destabilized systems. Such ionic strength‐induced face‐to‐face stacking of hematite nanoparticles was observed earlier for much thicker nanodisks as well [18].

**FIGURE 4:**
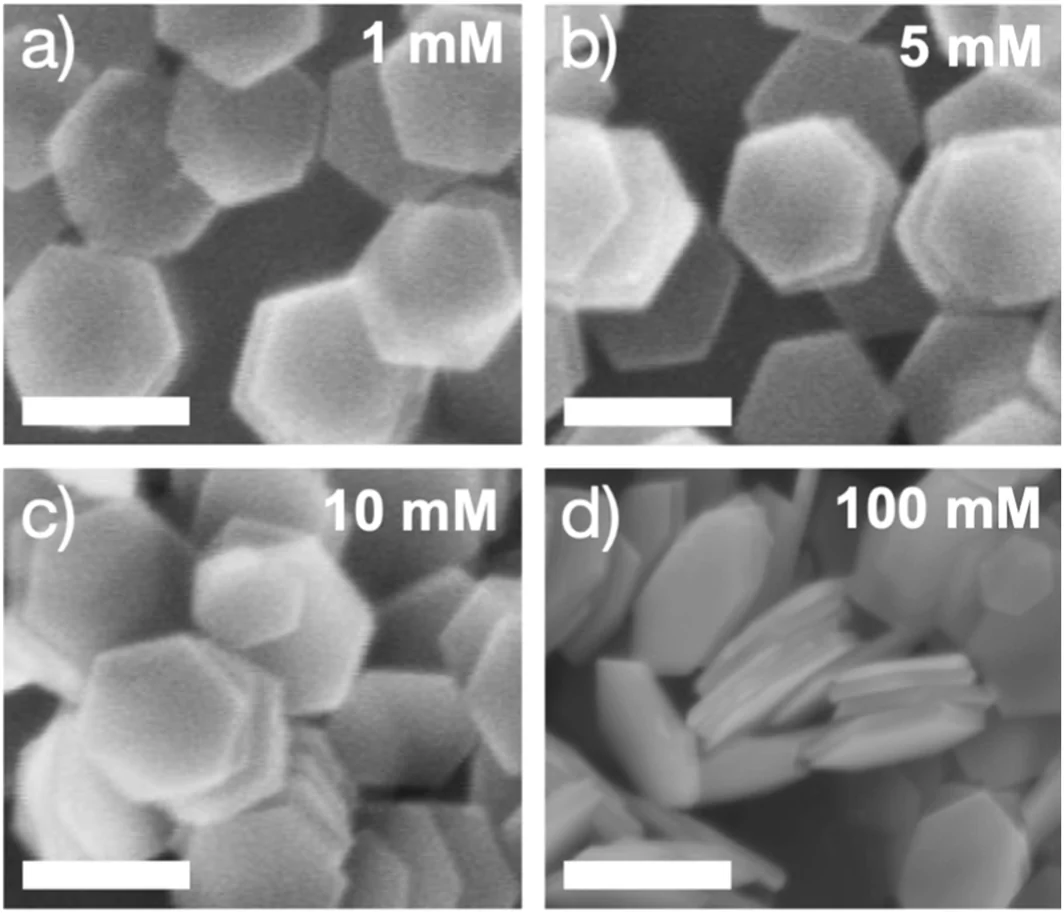
Scanning electron microscopy images of samples obtained after 30 minutes at 1, 5, 10, and 100 mM KNO_3_ concentration. Scale bar: 200 nm.

To gain a deeper insight into the stack formation related optical changes, simulation of the optical spectra of platelet stacks containing different numbers of platelets has been performed. As shown in Figure 5a, the simulation can capture the experimentally observed trend, that is, the increase of the relative extinction of the low energy peak around 575 nm with increasing number of particles in the stack compared to the main peak of the individual platelet at 450 nm. From the separate contributions of scattering and absorption (Supporting Information S1: Figure S6) it is clear that absorption of the growing stack scales roughly linearly with the number of particles forming the stack, while a pronounced scattering peak evolves at 575 nm. Hence, the experimentally observed spectral evolution can be assigned to the increased scattering at this wavelength. The electromagnetic near‐field maps and the corresponding field‐vectors obtained for a platelet monomer (Figure 5b, top row) are consistent with decomposed spectra shown earlier in Figure 2d. At 575 nm (marked by blue circle in Figure 5a), the electric near‐field shows the typical pattern of a dipole, which is not surprising as the main contribution to the overall scattering at this wavelength is related to the ED mode. However, there is also a minor contribution from the magnetic mode, which is consistent with the circular E‐field vector pattern inside the particle. Nevertheless this mode is very weak, and the local electric field pattern is dominated by the ED. As the number of the stacked particles increases, there is a gradual enhancement of both the electric and magnetic modes, resulting in a local field‐distribution clearly displaying the characteristic patterns of both an ED and MD (Supporting Information S1: Figure S7). For the stack consisting of 5 platelets (Figure 5b lower panel), the local electric field inside the particle obtained at 600 nm (red circle in the spectrum) shows the typical pattern characteristic of the MD mode, together with the prototypical circular E‐vector field [19]. Note that the electric field distribution is shifted along ‘*k*’ due to retardation. The pronounced magnetic field compared to the platelet monomer is also obvious. It has to be emphasized that both the electric and magnetic field patterns develop as the result of an in‐plane excitation of the platelet, and while the ED mode also lies in the plane, the corresponding MD mode is oriented out of plane.

**FIGURE 5:**
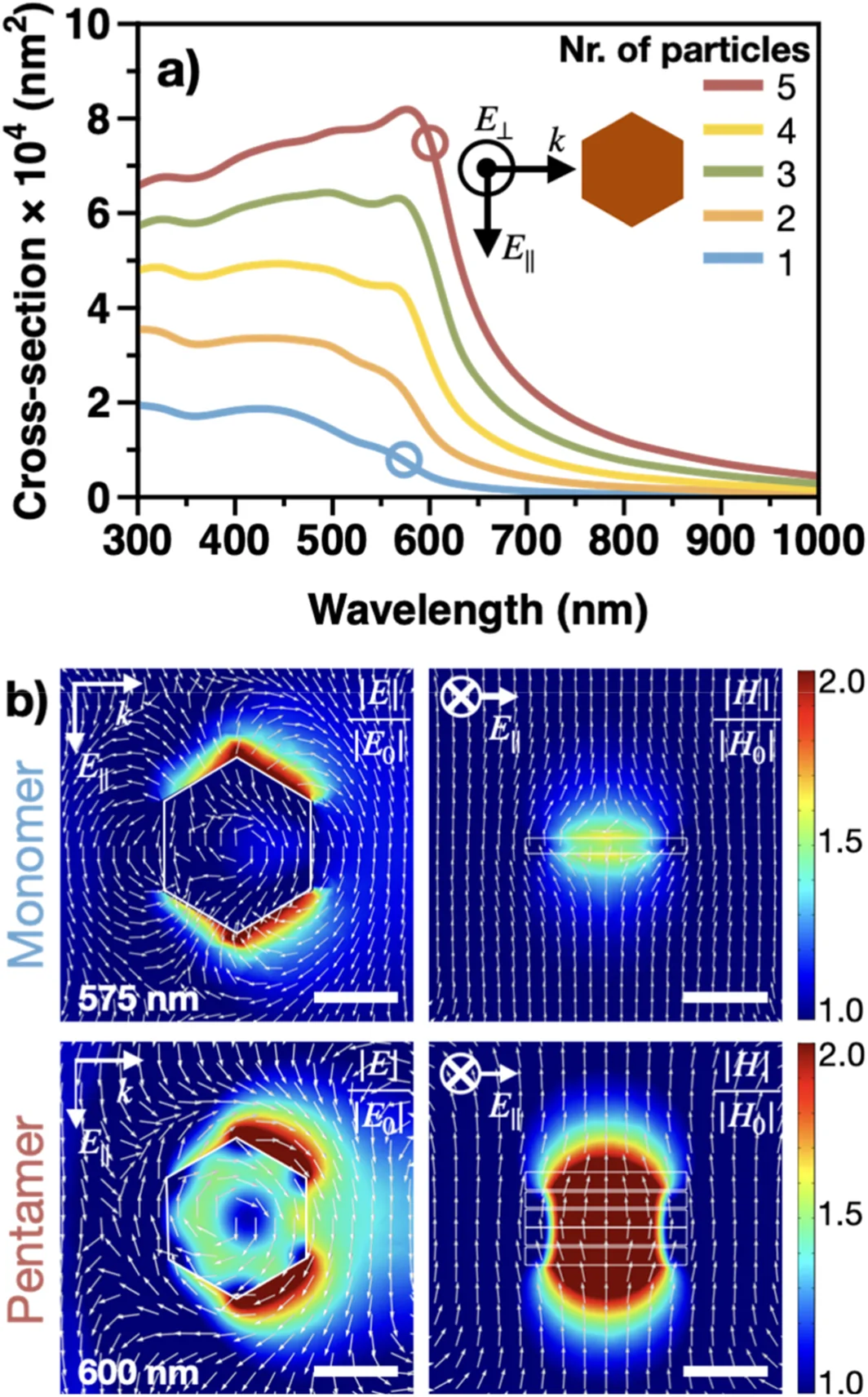
Simulated optical spectra of nanoplatelet stacks containing different numbers of particles (a). The spectra are averaged for the in‐plane and out‐of‐plane excitations. Electric (left) and magnetic (right) field distribution and normalized field vectors of the monomer (top) and pentamer (bottom) structures obtained at 575 and 600 nm, respectively, for the in‐plane excitation case (b). Scale bar: 100 nm.

When looking at the separate contribution of the ED and MD modes to the overall scattering, an important aspect of the stacking can be captured (Figure 6). Compared to MD, the ED contribution to the scattering is in general stronger as hematite is only a moderate refractive index material. The ED mode scales fairly linear with the number of particles, whereas the MD mode in contrast is siginificantly enhanced and provides a significant contribution to the overall scattering as the number of particles is increased. The observed wavelength‐modulation of both the ED and MD spectra can be ascribed to the characteristics of the dielectric function of hematite, as these are absent for a hypothetical, constant refractive index case (*n* = 3, *k* = 0; Supporting Information S1: Figure S8). It has to be emphasized that with increasing particle number, no characteristic peak splitting is observed that would indicate strong intraparticle interference or coupling of the electric and magnetic dipole modes [20, 21]. This is also supported by the fact that the calculated spectra are virtually independent on the particle–particle separation below the value of 10 nm (Supporting Information S1: Figure S9). Nevertheless, because the electric and magnetic modes overlap energetically and spatially there is still a possibility for some interaction based on the electric fields associated with these modes. This has been investigated by analyzing the phase and the relative phase difference of the dipole modes (Supporting Information S1: Figure S10) but no significant interaction between the ED and MD modes can be concluded [22]. Rather, the optical behavior of the self‐assembled particle stacks resemble that of a single, pillar‐like hematite nanoparticle with the corresponding height (Supporting Information S1: Figure S11). This also implies that the self‐assembly related stacking of the nanoplatelets leads to the formation of a Mie‐scatterer object, which otherwise not attainable directly in a wet‐chemical synthesis procedure. In addition, due to the vertical stacking‐determined co‐alignment of the individual platelets' magnetic dipole mode and the collective mode, Fano‐like interference, as observed earlier, for example, for 2D dielectric particle assemblies, can be ruled out [23, 24]. It has to be noted that during simulation an idealized structure consisting of equally sized and oriented platelets were considered. Nevertheless, rotational alignment has only negligible impact on the spectra, whereas a nonuniform platelet size can lead to a slight decrease of the relative scattering peak intensity at 575 nm associated with the electric and magnetic dipoles (Supporting Information S1: Figure S12). It should be emphasized that based on calculations the vertical arrangement of the platelets in the stack is required to allow the development of the collective magnetic dipole mode associated with the 575 nm peak because this is virtually absent for, for example, a planar aggregate structure (Supporting Information S1: Figure S13).

**FIGURE 6:**
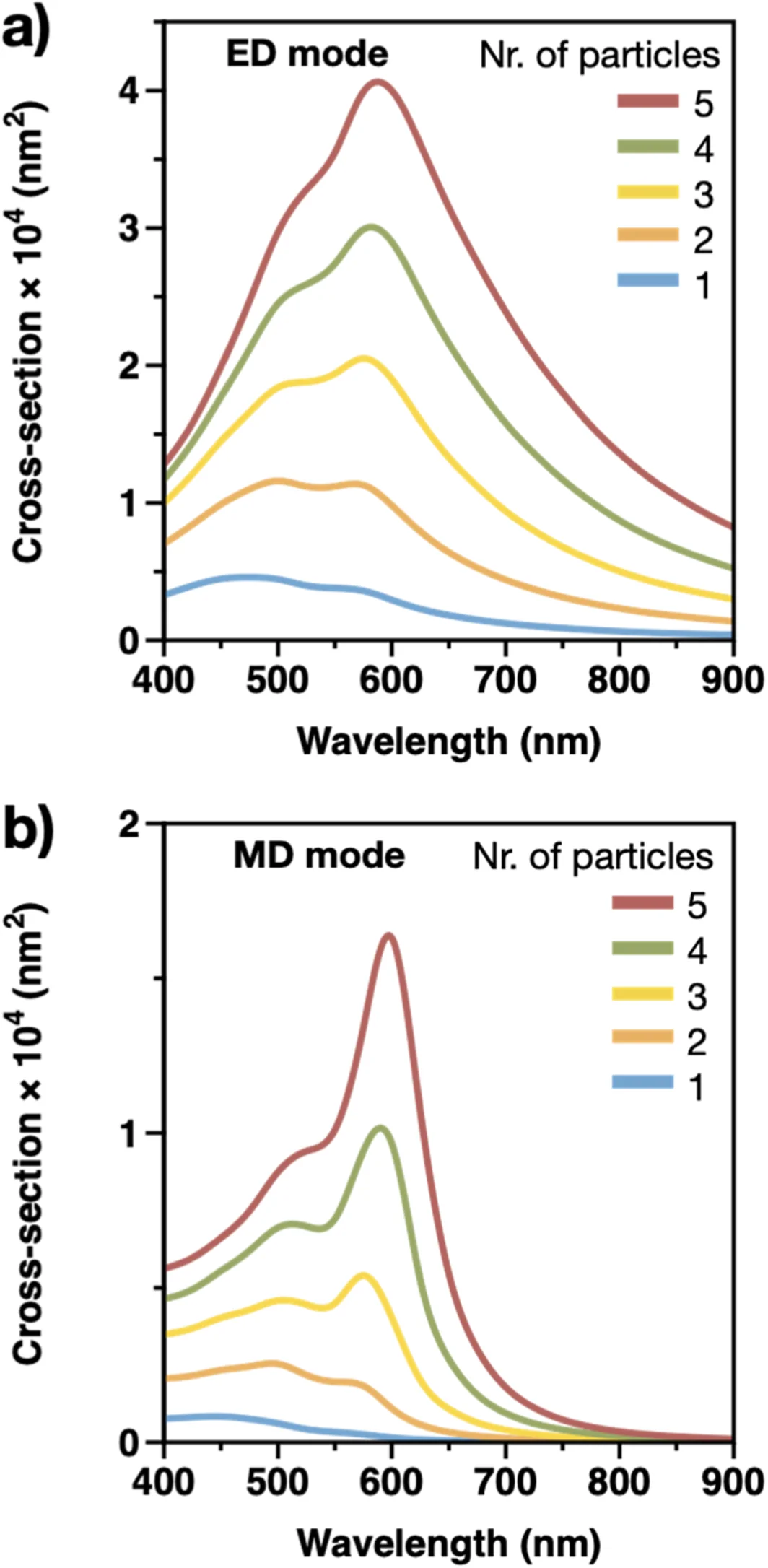
Development of the calculated collective electric (a) and magnetic (b) dipole mode scattering in a nanoplatelet stack with increasing number of particles (averaged for the in‐plane and out‐of‐plane excitations).

The change of the respective dipole mode contributions with increasing particle number is summarized in Figure 7. Both the ED and MD modes are enhanced as they deviate in the positive direction from the simple additivity of the single plate's corresponding mode (dashed line). Although the increase of the ED mode's contribution remains moderate, the MD mode is significantly enhanced, indicating that the relative contribution to the spectral changes observed around 600 nm in the experiments can be primarily ascribed to the emergence of a strong collective MD mode.

**FIGURE 7:**
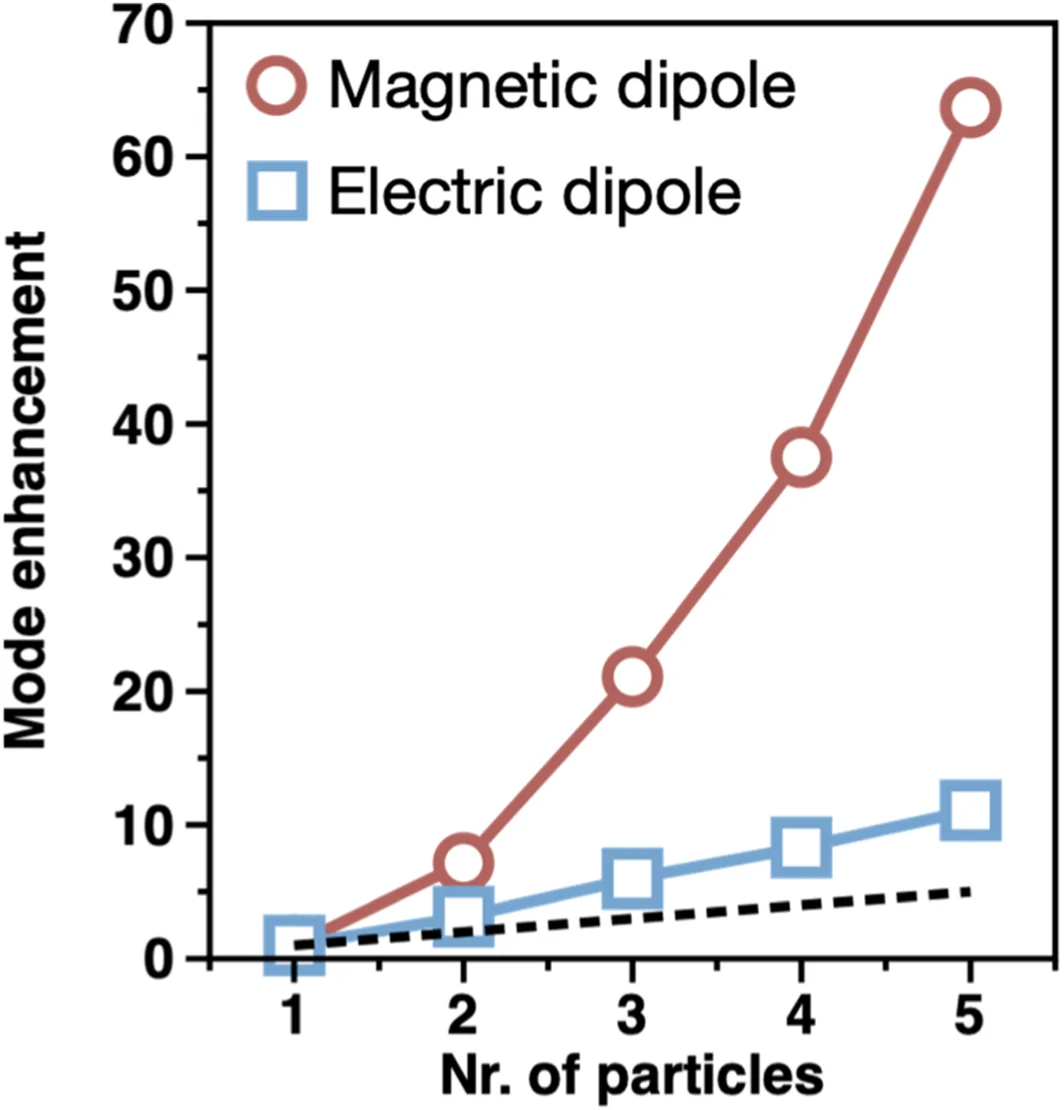
Calculated enhancement of the collective magnetic and electric dipole modes in the particle stacks compared to the simple additivity (dashed line) as a function of the number of particles. The enhancement is obtained by normalizing the corresponding mode contribution (intensity at the modes' characteristic wavelength) with that of a single platelet.

To corroborate the findings of the optical simulations, correlative single entity dark‐field scattering spectroscopy/scanning electron microscopy experiments have been carried out. Individual nanoplatelets and particle stacks have been identified and imaged in SEM, which have been subsequently located and measured in a dark‐field optical microscope‐based imaging spectroscopy system. This approach has been used earlier to capture the size related scattering properties of individual Si, TiO_2_, Se, Cu_2_O, and other nanoparticle types [9, 19, 25, 26, 27]. The measured spectra of the individual objects show a clear intensity and wavelength dependence on the number of platelets in the stack (Figure 8a). Due to the small thickness of the individual platelets (ca. 20 nm) and the stack structure unambiguous determination of the exact number of the platelets in the stacks is not possible. Nevertheless the increasing overall scattering intensity implies an increasing platelet number, which is accompanied by the relative increase of the scattering peak between 550 and 600 nm, leading to a dual‐peak spectrum for the investigated stacks. As in the given dark‐field measurement the particles are supported by a substrate, illumination is at oblique angle, and the collection of the scattered light is detected in angular directions restricted by the numerical aperture of the objective, additional simulations have been performed to account for this measurement‐specific constraints (see Experimental Section for details) [28]. The simulated spectra show a good agreement with the measured spectra, and effectively capture the experimentally observed intensity scaling of the scattering spectrum and peak evolution (Figure 8b). Based on the separate contribution of the MD and ED modes to the scattering (Supporting Information S1: Figure S14), the ED mode is more than two times stronger than the MD mode, just as it was found earlier in the solution‐based simulations. The contribution of the MD mode to the shorter wavelength scattering peak, however, is more pronounced and results in the evolution of the dual‐peak spectrum as the number of platelets in the stack increases. It has to be pointed out that in the present system the magnetic mode becomes only pronounced as the stack grows; for a single platelet, its contribution to the total scattering is marginal. Hence, as the platelets are assembled in larger structure, the collective mode turns them into a much more effective Mie‐scatterer for the visible wavelength range.

**FIGURE 8:**
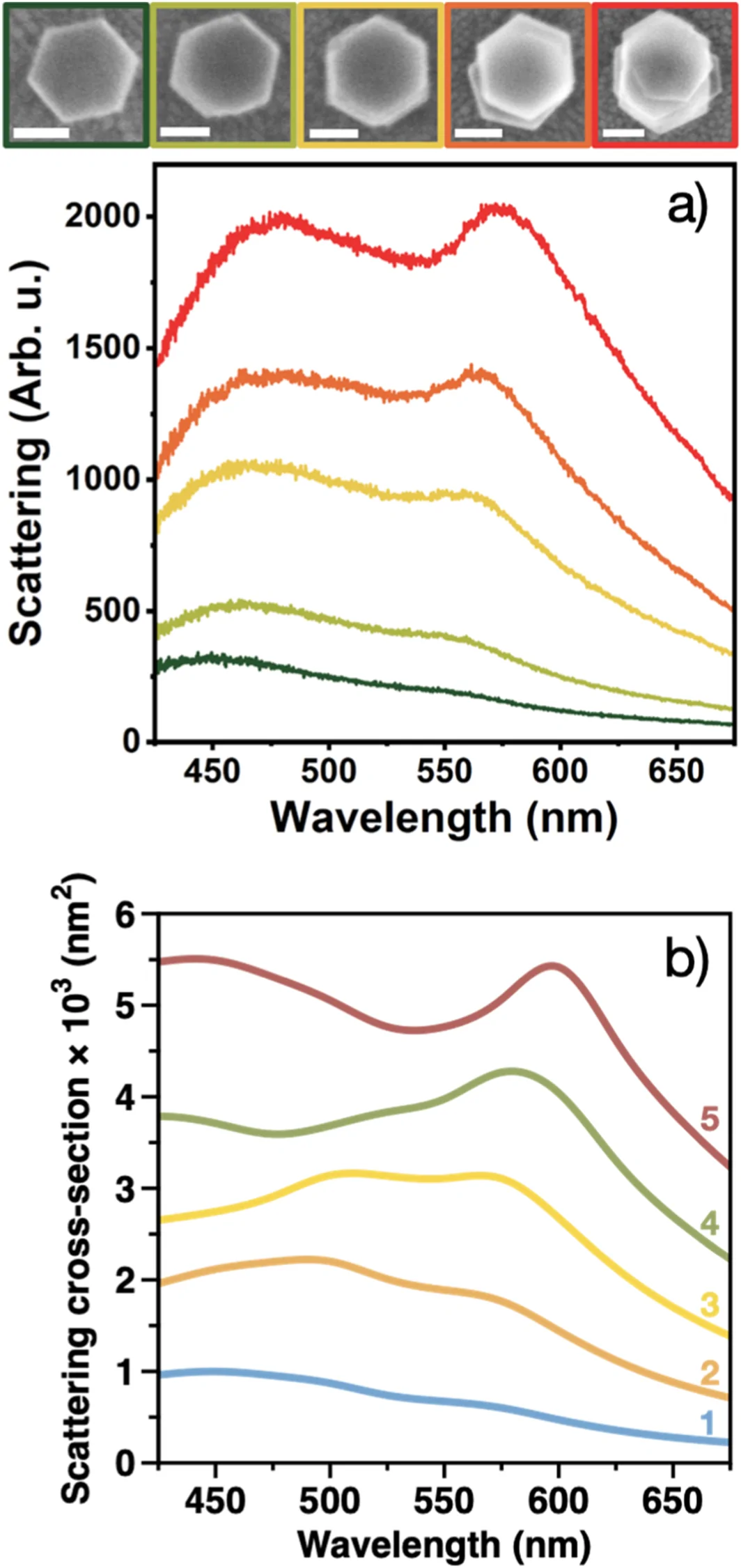
Measured single individual platelet and stack scattering spectra, with the corresponding SEM images shown in the upper panel (a, Scale bars: 100 nm). Simulated optical scattering spectra of the substrate supported objects with an increasing number of particles in the stack, averaged for the TE and TM excitations (b).

## 3 Conclusion

The self‐assembly of hematite nanoplatelets into stacked structures induces changes in their optical extinction spectra. When the platelet‐like particles lose their colloidal stability and assemble into vertical stacks, a peak evolution around 575 nm is observed in their ensemble extinction spectra. Optical simulations have shown that this can be explained by the self‐assembly enabled evolution of a collective magnetic dipole mode. Although the individual hematite nanoplatelets show marginal magnetic dipole moment, the self‐assembled Mie‐scatterer stacks feature a relatively sharp magnetic dipole scattering peak compared to the electric dipole mode, responsible for the observed peak evolution. Although there is both a spatial and energetic overlap between the electric and magnetic dipole modes in the stacks, no clear indication for a magnetoelectric coupling is observed. Instead, the spectral shape evolution can be explained by the combination of hematite's dielectric function and the well‐defined, largely enhanced collective magnetic dipole mode. The findings are also corroborated by correlative single‐object scattering spectroscopy/electron microscopy, clearly showing the appearance and increase of the scattering peak related to the enhanced magnetic dipole mode with increasing number of hematite platelets in the stack. The results demonstrate that Mie‐scattering particles, which cannot be directly obtained via a solution‐based synthetic approach, can be formed through a self‐assembly route. This offers a promising strategy for modulating the Mie scattering of nanoparticles, highlighting the potential for mode engineering in these nanophotonic structures similarly to their plasmonic counterparts.

## 4 Experimental Section

Chemicals: Iron(III) chloride (FeCl_3_, 97%), sodium acetate (NaAc, 99%), potassium nitrate (KNO_3_, 99.0%), ethanol (EtOH, 99.95%), ultrapure water (18.2 MΩ·cm resistivity).

Nanoplatelet synthesis and assembly: Hematite nanoplatelets (HNPs) were synthetized by the method described in Ref [29]. Briefly, FeCl_3_ (0.4055 g) was dissolved in EtOH (25 mL), then transferred to a 100 mL teflon‐lined stainless steel autoclave. Ultrapure water (1.75 mL) and solid NaAc (2.000 g) was added and the mixture was homogenised. The autoclave was heated in an oven (Memmert UN55) at 180°C for 12 h. Upon cooling to room temperature, the supernatant was removed, the particles were redispersed in ultrapure water then left overnight for sedimentation. This process was repeated one more time and the resulting reddish‐brown suspension was sonicated for 10 minutes (after which no sedimentation was observed), then stored in a refrigerator.

For the ensemble characterization HNP suspension (5 μL) and ultrapure water (995 μL) was mixed and sonicated for 10 minutes, then transferred to a semi‐micro cuvette. For studying the electrolyte‐induced assembly (stacking) of the HNPs, HNP suspension (5 μL) and ultrapure water (895 μL) was mixed, sonicated for 10 minutes and transferred to a semi‐micro cuvette, then KNO_3_ (100 μL) solution was added so that the final KNO_3_ concentration was 1, 5, 10, or 100 mM.

Ensemble characterization: Extinction spectra were recorded in the 300–1000 nm range with a Shimadzu UV‐3600i UV‐Vis‐NIR spectrophotometer. For the time‐dependent spectroscopy, measurement was started immediately after the addition of KNO_3_ and 40 extinction spectra were recorded at five‐minute intervals (200 minutes in total).

Dynamic light scattering measurements were performed with a Malvern ZetaSizer Nano ZS device to determine the size distribution of the particles. The time‐dependent measurements started immediately after the addition of KNO_3_, and 40 size distribution functions were measured at five‐minute intervals (200 minutes total). For electrophoretic mobility measurements, KNO_3_‐containing particle suspensions were created with the above described method with KNO_3_ concentration between 0.01 and 3 mM and measured in folded capillary cells (DTS‐1070).

Structural characterization: For scanning electron microscopy (SEM) HNPs were deposited onto silicon wafers via drop casting from a diluted aqueous suspension and dried under ambient conditions. SEM images were recorded at 5 keV accelerating voltage with a Zeiss LEO 1540‐XB device. A Bruker AXS D8 Discover X‐ray diffractometer equipped with Göbel‐mirror and scintillation detector using Cu Kα (*λ* = 1.5406 Å) radiation was used to measure the diffractogram of the synthesized nanoplatelets. The X‐ray beam dimensions were 1 mm × 5 mm, the 2Θ step size was 0.05°, scan speed 0.15° min^−1^. The Diffrac.EVA program and the ICDD PDF database was used for phase identification.

Correlative microspectroscopy: Particles were deposited onto indium‐tin‐oxide (ITO)‐coated glass substrates (PGO GmbH) via spin‐coating method (2600 rpm, 30 s, 50 μL suspension). The substrates were cleaned prior to particle deposition using sonication (acetone, isopropanol, water, 15 min each) and air‐plasma treated for 1 min. Individual HNPs and aggregates (stacks) with different numbers of platelets were identified in scanning electron microscope. Optical scattering spectra of exactly the same individual particles and stacks have been measured with a laboratory‐build microspectroscopy instrument. The setup is built around an Olympus BX51 upright microscope equipped with an Olympus 50× MPlanFLN (NA = 0.8) dark‐field objective and a 100 W Olympus U‐LH100IR IR‐enhanced illumination source. A PI P‐545.xR8S XYZ piezo‐stage is used for precise sample positioning and focusing. The microscopy image is relayed onto the entrance slit of an imaging spectrometer (Princeton Instruments Isoplane SCT320 with a PIXIS 400 BRX camera operated at −70°C). A laboratory‐developed software written in Labview is used to optimize the scatterers' position and to take the average of 7 spectra with 1 s exposure time each. The spectra are automatically corrected with the optical transfer function of the setup (calibration performed by a NIST traceable wavelength and intensity calibration lamp from Princeton Instruments).

Optical simulations: The single particle and stack optical properties have been obtained using the Wave‐Optics Module in COMSOL Multiphysics 5.3a. The dielectric data (*n*, *k*) for hematite was obtained from ref. [30]. The hematite nanoparticle was simulated as 190 nm (tip‐to‐tip) × 20 nm hexagonal platelet. In the stacks the gap between the particles was set to 2 nm. Two main cases were considered: (i) particle/stack embedded in a homogeneous environment or (ii) on a substrate.

- The embedding medium was assumed to be water (*n* = 1.33). The objects were placed at the center of a 1 μm spherical volume, with an additional 200 nm thick perfectly matched layer divided into 5 concentric layers. Planewave excitation was performed both for the in‐plane and the out‐of‐plane situations, with the propagation direction being in the plane of the platelet. The scattering, absorption and extinction cross‐section of these two excitation modes have been averaged. Mode decomposition of the scattering was performed using the formulae provided in ref. [31], which can be conveniently adapted for finite‐element methods. The contributions of the ED, MD, EQ, and MQ were obtained. To investigate the phase relations the complex electric and magnetic fields have been integrated over the particle/stack volume to get the net complex electric and magnetic moments and their argument has been calculated.
- For the substrate supported case, the same particle/stack was placed at the interface of air (*n* = 1) and a dielectric semi‐infinite slab (*n* = 1.4). The angle of incidence was set to 65° corresponding to the average illumination angle of the dark‐field microscope objective used during the experiments. TE and TM excitation polarization has been defined with either the electric or magnetic component of the excitation being perpendicular to the plane of incidence and the other field components set to 0. The simulation was run first in the absence of the particle/stack, by simply overriding its dielectric function with that of the air domain. This resulted in a solution for the local field distribution after partial reflection at the air/substrate interface, which was used as the excitation field to calculate the particle/stack optical properties. The actual scattering spectra were obtained performing a surface integral of the time‐averaged power outflow of the relative (scattered) electric field on a spherical cap located 300 nm above the particle/stack. The cap had a dimension to match the numerical aperture of the collection cone of the microscope objective (*NA* = 0.8) used during the actual experiments. The obtained quantities were normalized by the input excitation power.

## Author Contributions

**Péter Márton:** investigation, visualization, writing – original draft, validation, methodology, writing – review and editing. **Dániel Zámbó:** visualization, investigation, validation, methodology, writing – review and editing, writing – original draft. **András Deák:** conceptualization, methodology, writing – review and editing, writing – original draft, software, supervision, project administration, funding acquisition, visualization, investigation, validation, data curation.

## Funding

OTKA FK 142148, NKKP Advanced 153064, EKÖP‐25‐4‐I‐BME‐132.

## Conflicts of Interest

The authors declare no conflicts of interest.

## Supporting information


Supporting Information S1

## Data Availability

The data that support the findings of this study are openly available in HUN‐REN ARP at https://repo.researchdata.hu/privateurl.xhtml?token=69ae2a96‐b01b‐4a94‐a11f‐b6433bd629bb.
