# Supplementary material for: Self‐Assembly Enabled Mie‐Scattering in Hematite Nanoplatelet Stacks
Source: Nanophotonics. 2026 Aug 13;15(16):e70253. doi: 10.1002/nap2.70253 (PMC13672148; doi:10.1002/nap2.70253)
Supplement: Supplementary file 1 [file NAP2-15-e70253-s001.pdf]

Supporting Information

Self-assembly Enabled Mie-scattering in Hematite Nanoplatelet Stacks

*Péter Márton, Dániel Zámbó, and András Deák**

P. Márton, D. Zámbó, A. Deák

Photonics Department, HUN-REN Centre for Energy Research, Budapest, Hungary

P. Márton

Department of Physical Chemistry and Materials Science, Budapest University of Technology and Economics, Budapest, Hungary

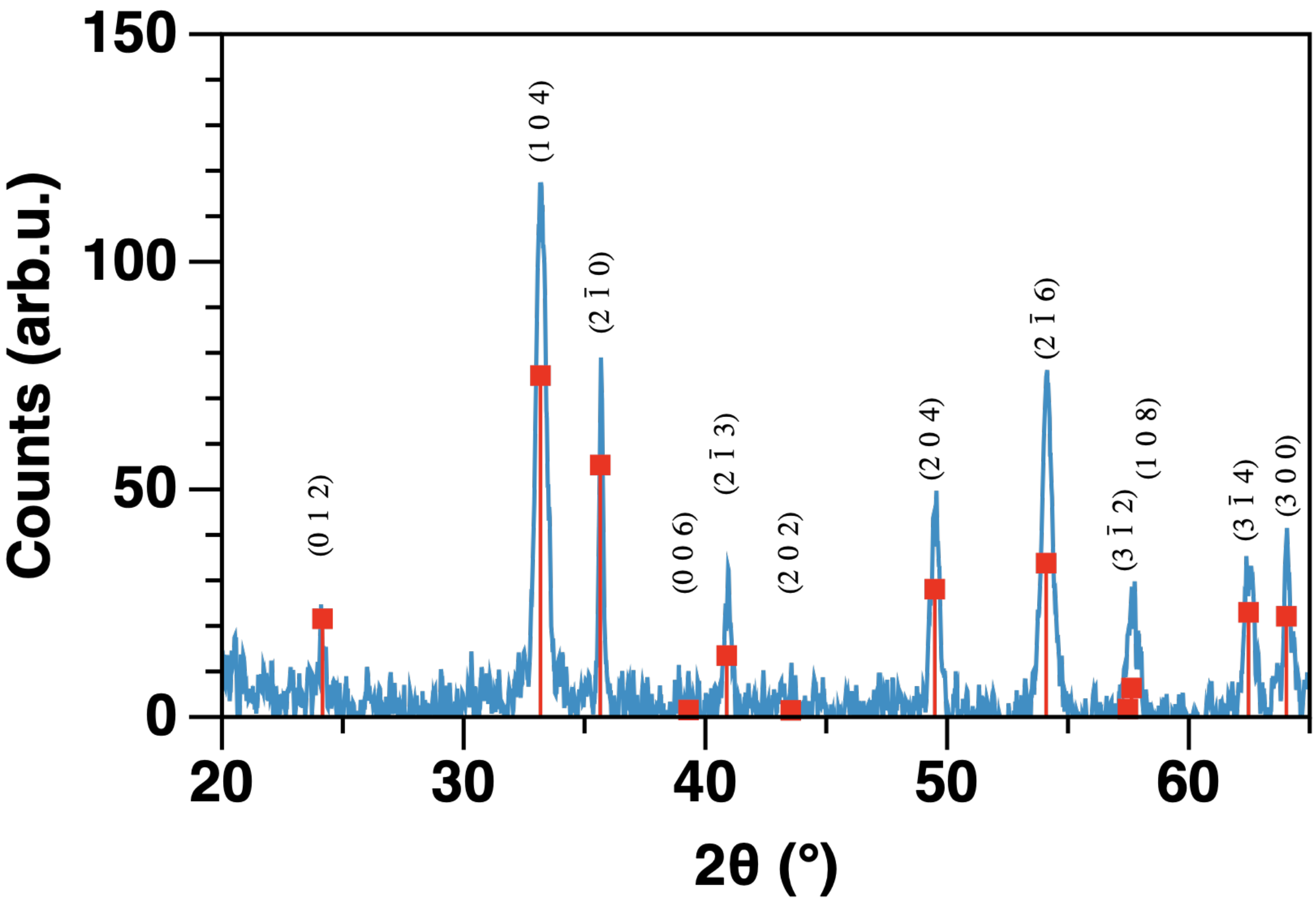

Figure S1. XRD diffractogram of the prepared nanoplatelets compared to the hematite 01-089-0599 reference patterns from the International Centre for Diffraction Data Powder Diffraction File. Indices correspond to the rhombohedral crystal structure of hematite described in hexagonal convention.

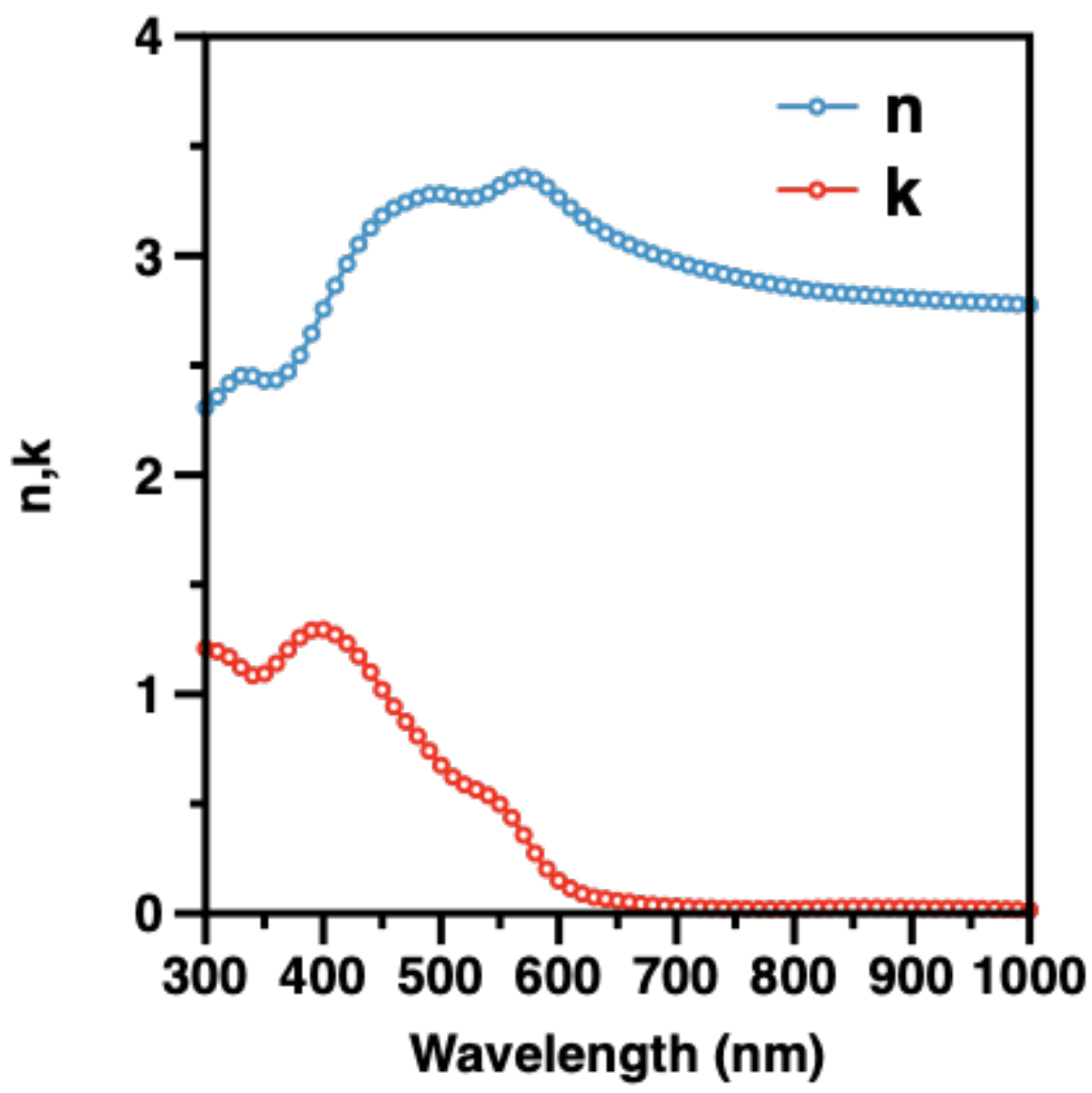

Figure S2. Real and imaginary parts of the refractive index of hematite from Ref. 1.

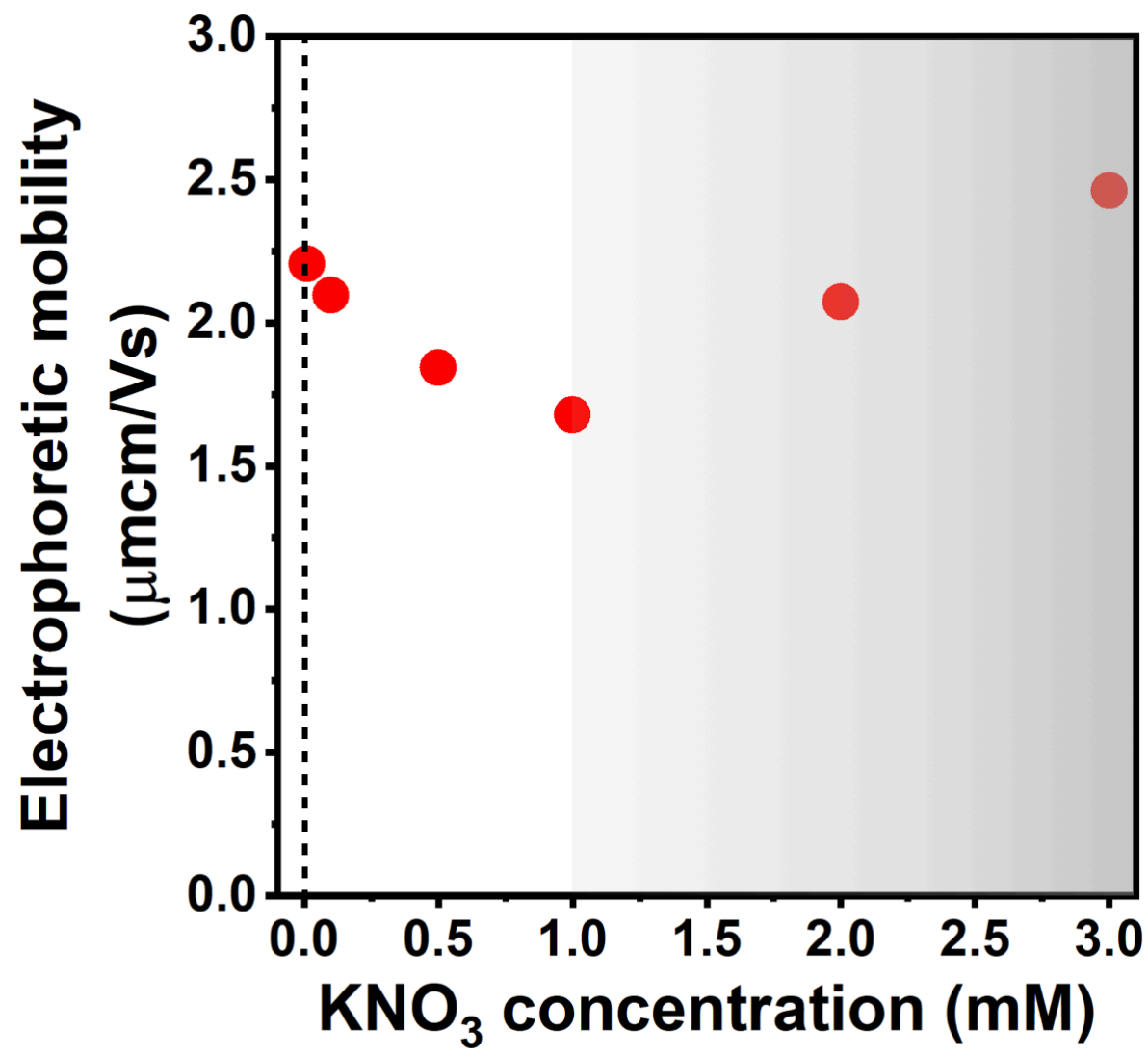

Figure S3. Electrophoretic mobility of the hematite nanoplatelets obtained in the presence of KNO_3 (concentration range: 10 uM - 3 mM). The shaded region indicates the KNO_3 concentration range, where particle aggregation takes place.

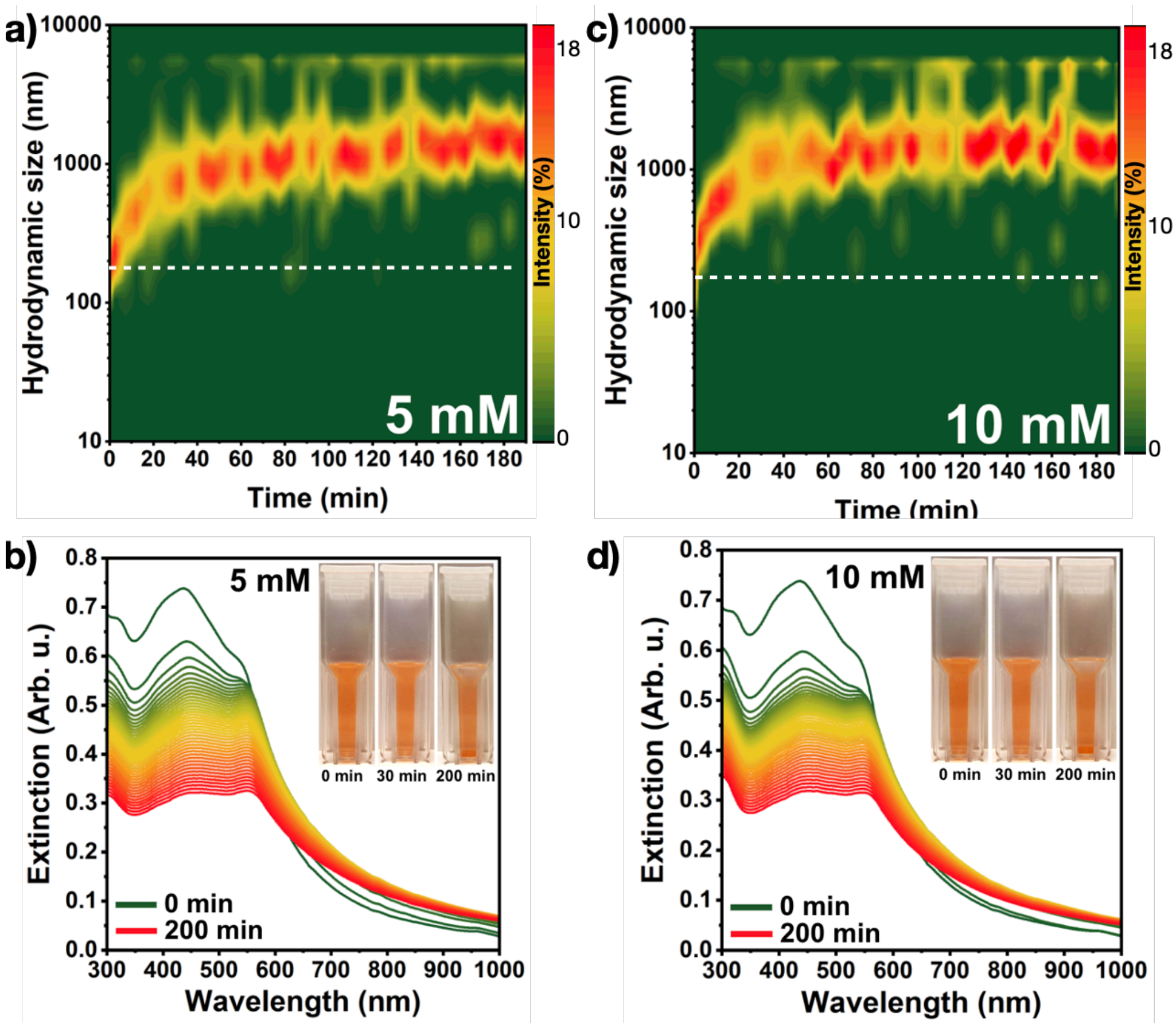

Figure S4. Time-dependent dynamic light scattering derived size distribution (top) and ensemble optical spectra (bottom) of the particles recorded at 5 mM (a,b) and 10 mM (c,d) KNO_3 concentrations. The dashed line in the DLS plots indicates the pristine nanoplatelet size.

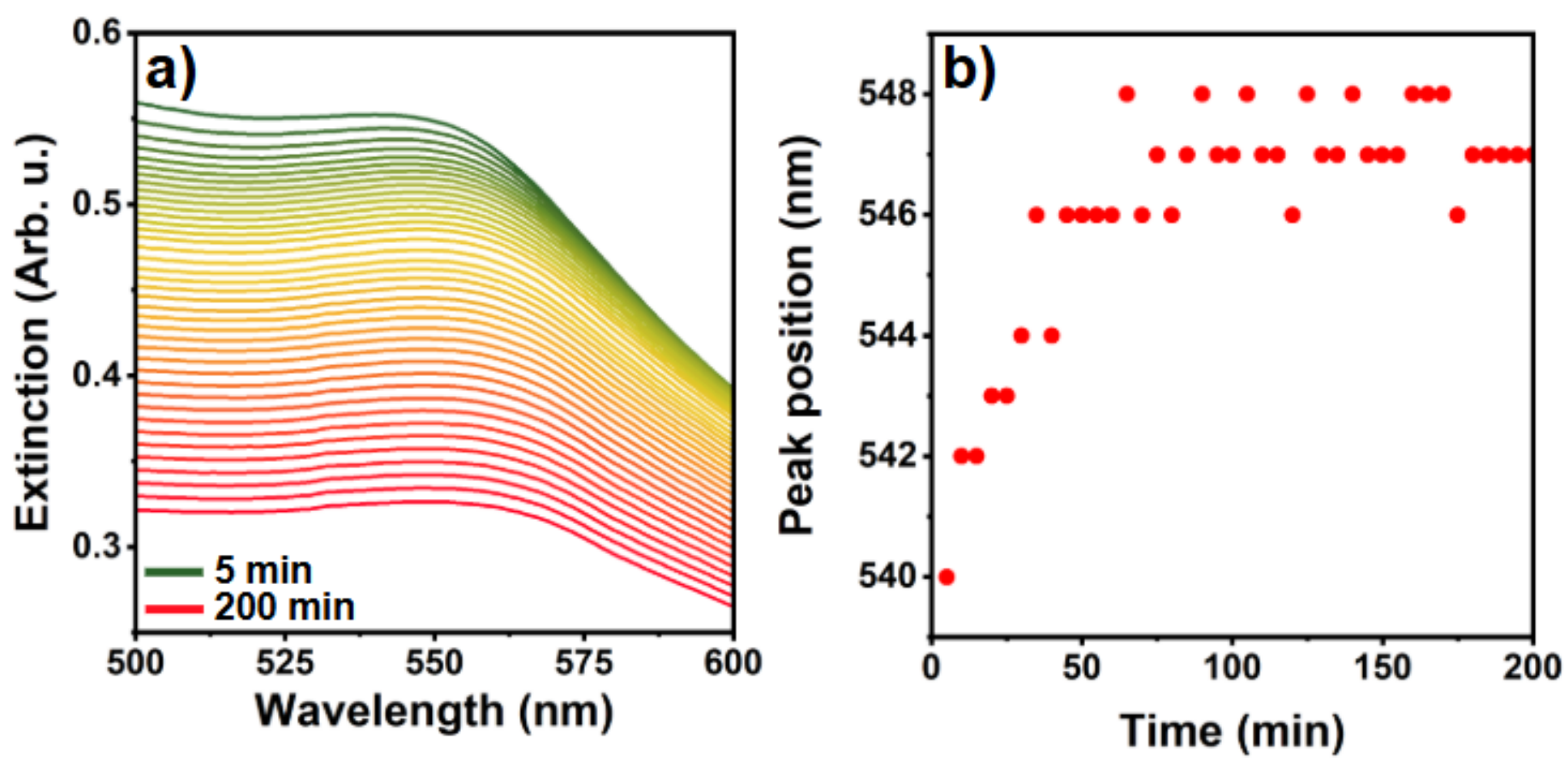

Figure S5. Magnified region of the ensemble spectra measured at 100 m KNO_3 around 550 nm (a) and the extracted wavelength position of the local maxim (the quantized character of the data is a result of the spectrometer's limited wavelength resolution) (b).

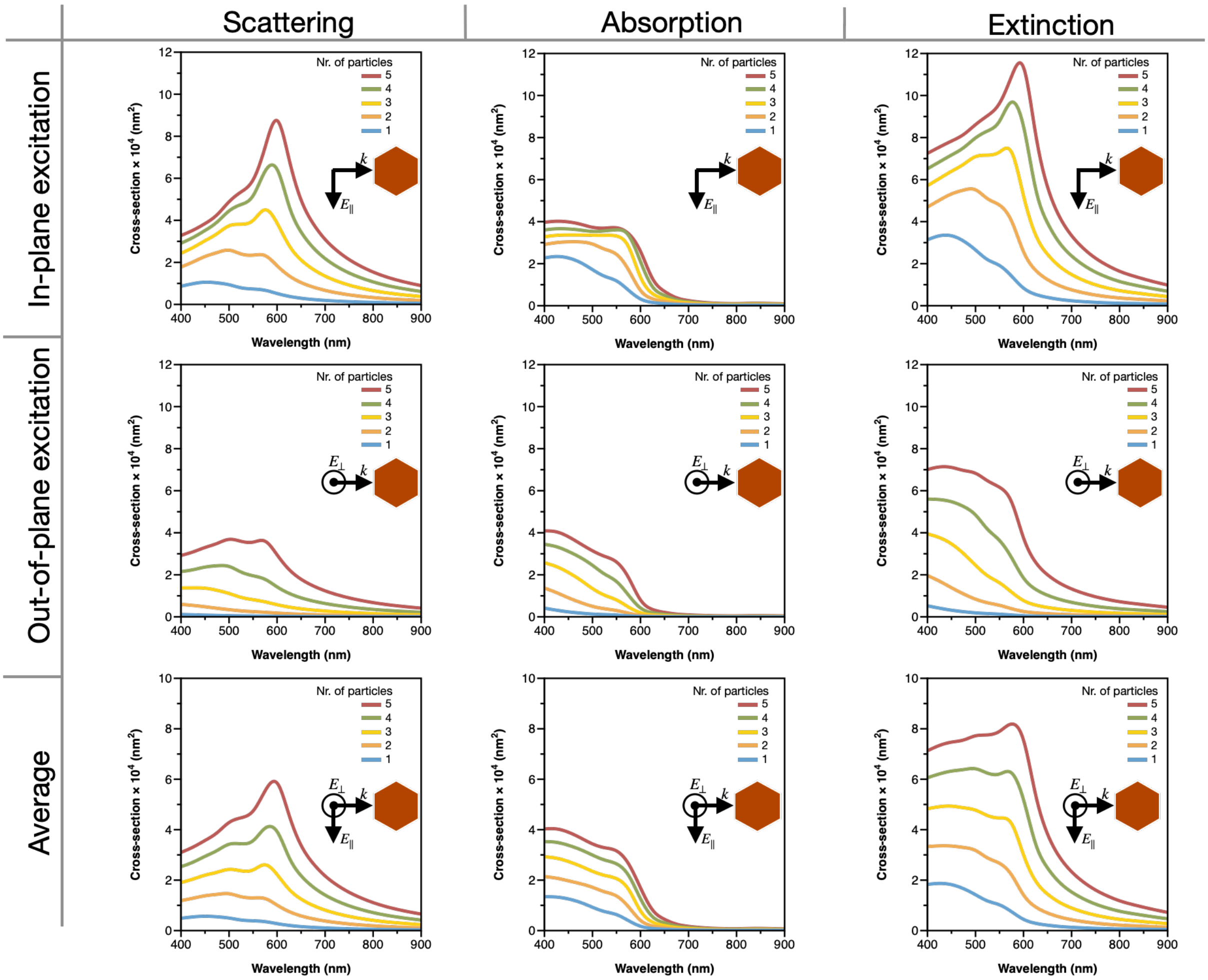

Figure S6. Simulated optical spectrum of the platelet stack with different numbers of particles in the assembly. The bottom row shows the average of the two excitations (in-plane and out of plane). The interparticle gap is 2 nm.

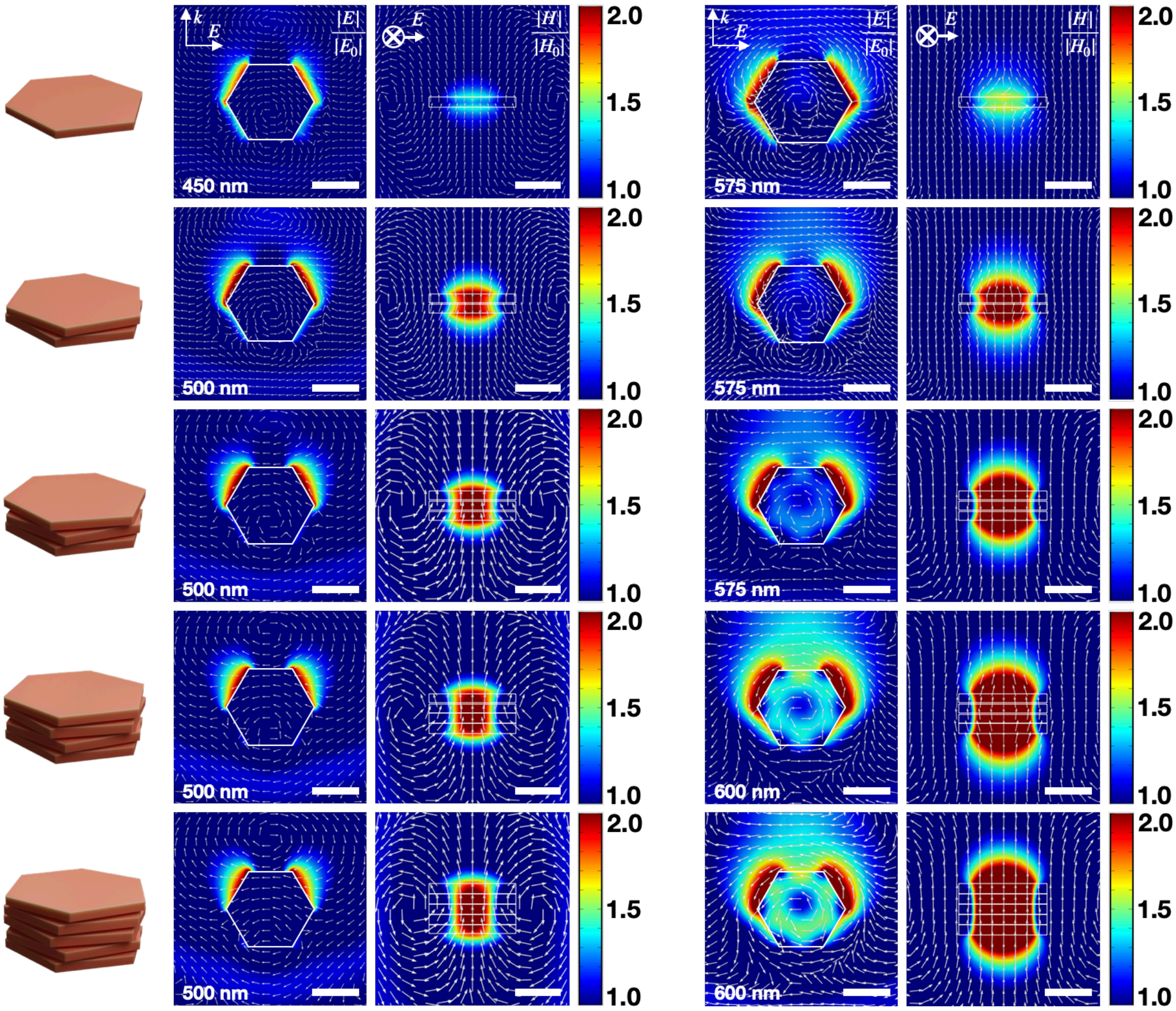

Figure S7. Local field-distribution in the stacks containing an increasing number (1-5) of hematite nanoplatelets in the stack, excited at two characteristic wavelengths: Left panel - short wavelength; Right panel - long wavelength (excitation wavelength highlighted in the figure). In each panel the left column shows the electric field, the right the magnetic field distribution. The field vectors are normalized for better visibility.

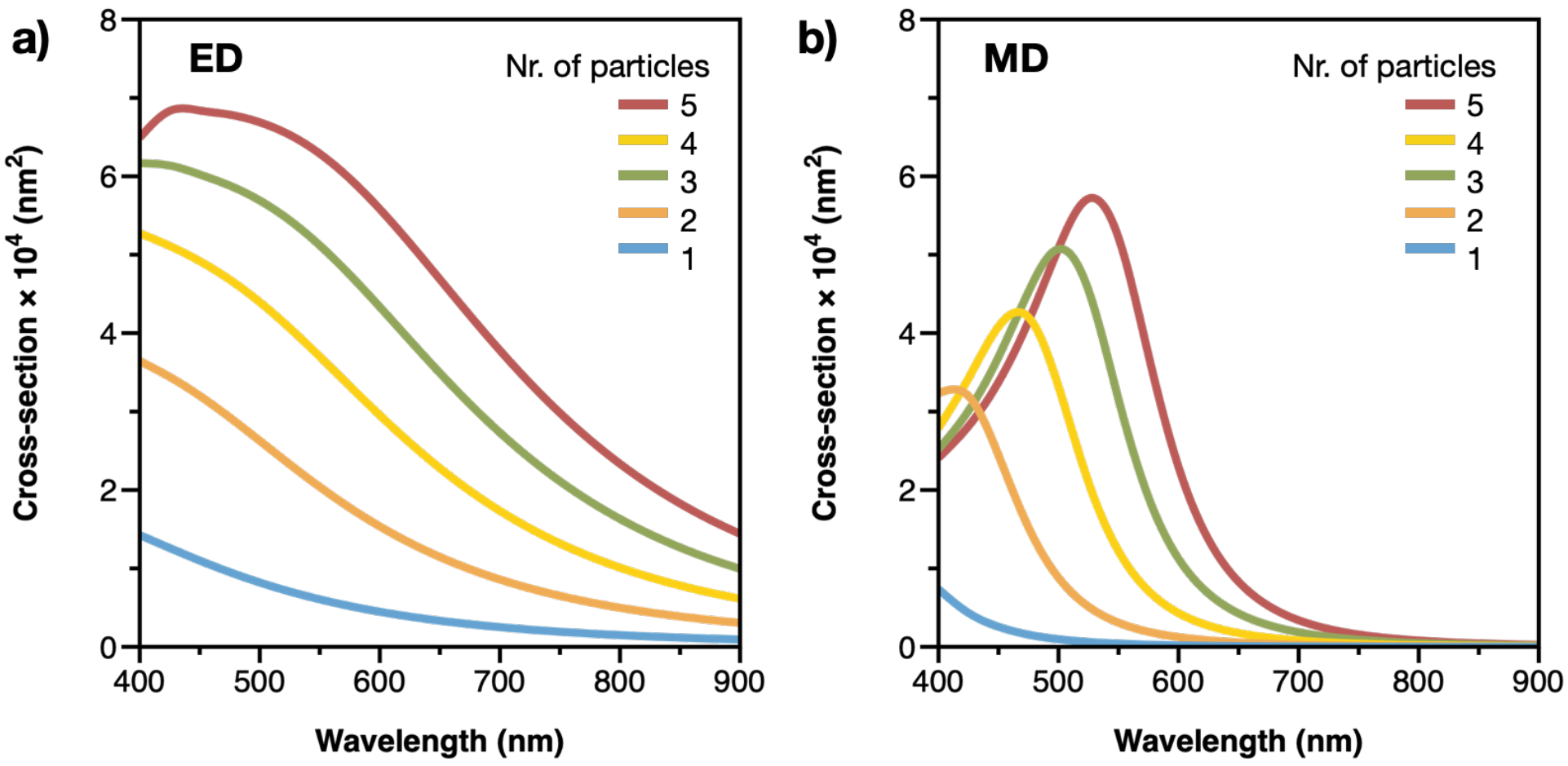

Figure S8. Calculated spectrum of the electric (ED) and magnetic (MD) dipole modes of stacks containing different numbers of nanoplatelets with constant refractive index (n=3; k=0) for the dominant, in-plane excitation case.

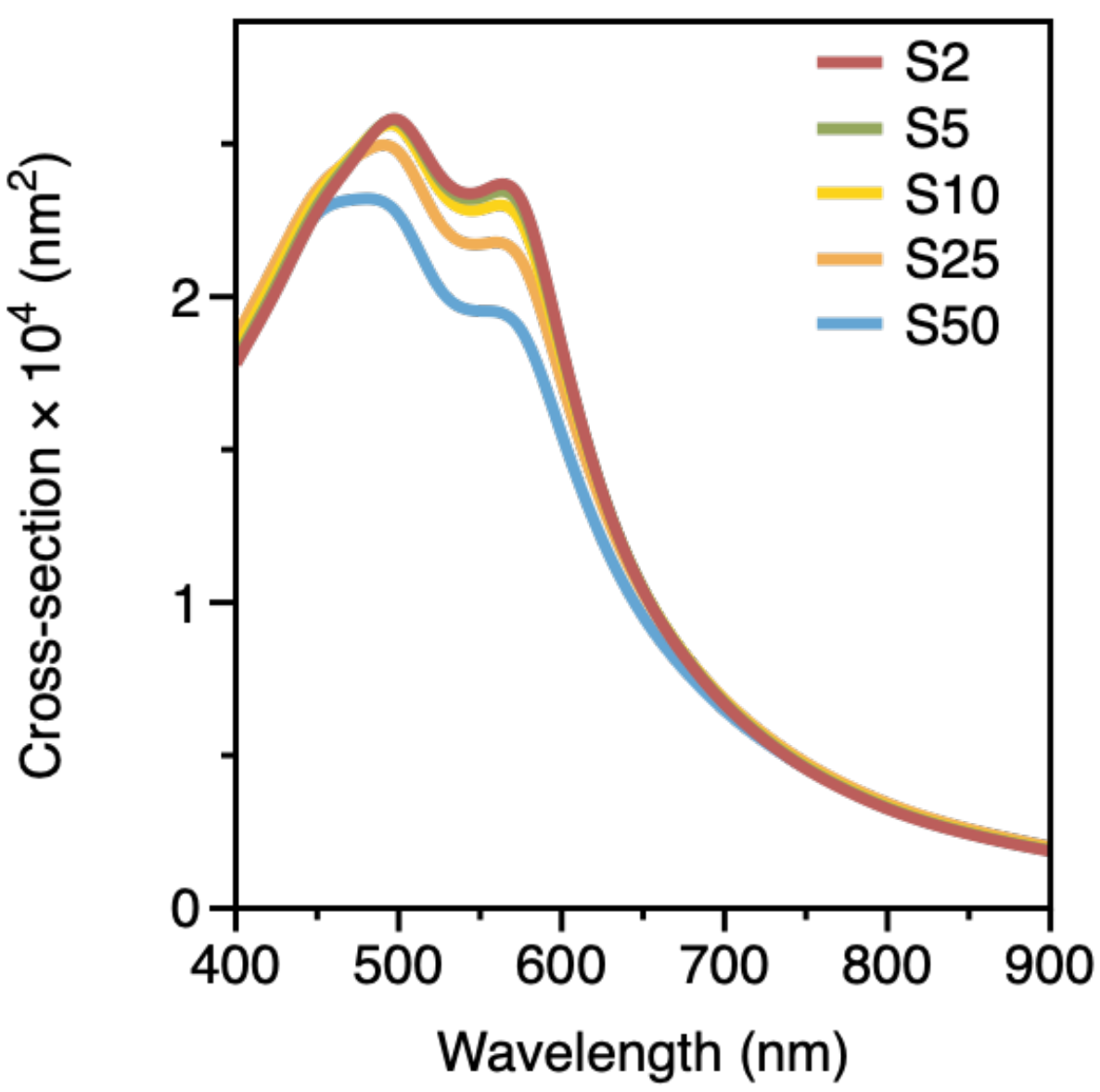

Figure S9. Simulated scattering spectra of a nanoplatelet dimer as a function of particle-particle separation.

For the phase analysis, the field components aligned with the principal axes of the corresponding dipole mode were evaluated: ED_z for the electric field (parallel to the exciting E-field, in the plane of the platelet), MD_y for the H-field (perpendicular to both the E-field and the propagation direction, out-of-plane); see inset in Figure S10b). At longer wavelength the phase of both the in-plane electric dipole (ED_z) and out-of-plane magnetic dipole (MD_y) modes is around zero, indicating that they oscillate independently in-phase with the external driving electric field as expected. In agreement with the broad character of the peaks, the transition to a π/2 phase lag at shorter wavelength is rather smooth. As the magnetic mode gains intensity with an increasing number of particles, the relative phase difference (Δφ) change around 600 nm becomes steeper. This, however, is a consequence of the MD mode intensity increase, leading to a more defined MD peak, rather than a real interference between the ED_z and MD_y modes. This is supported by the dimer spectra obtained for different particle-particle separations, showing no sign of mode splitting or appearance of new peaks in the spectra (**Figure S9**).

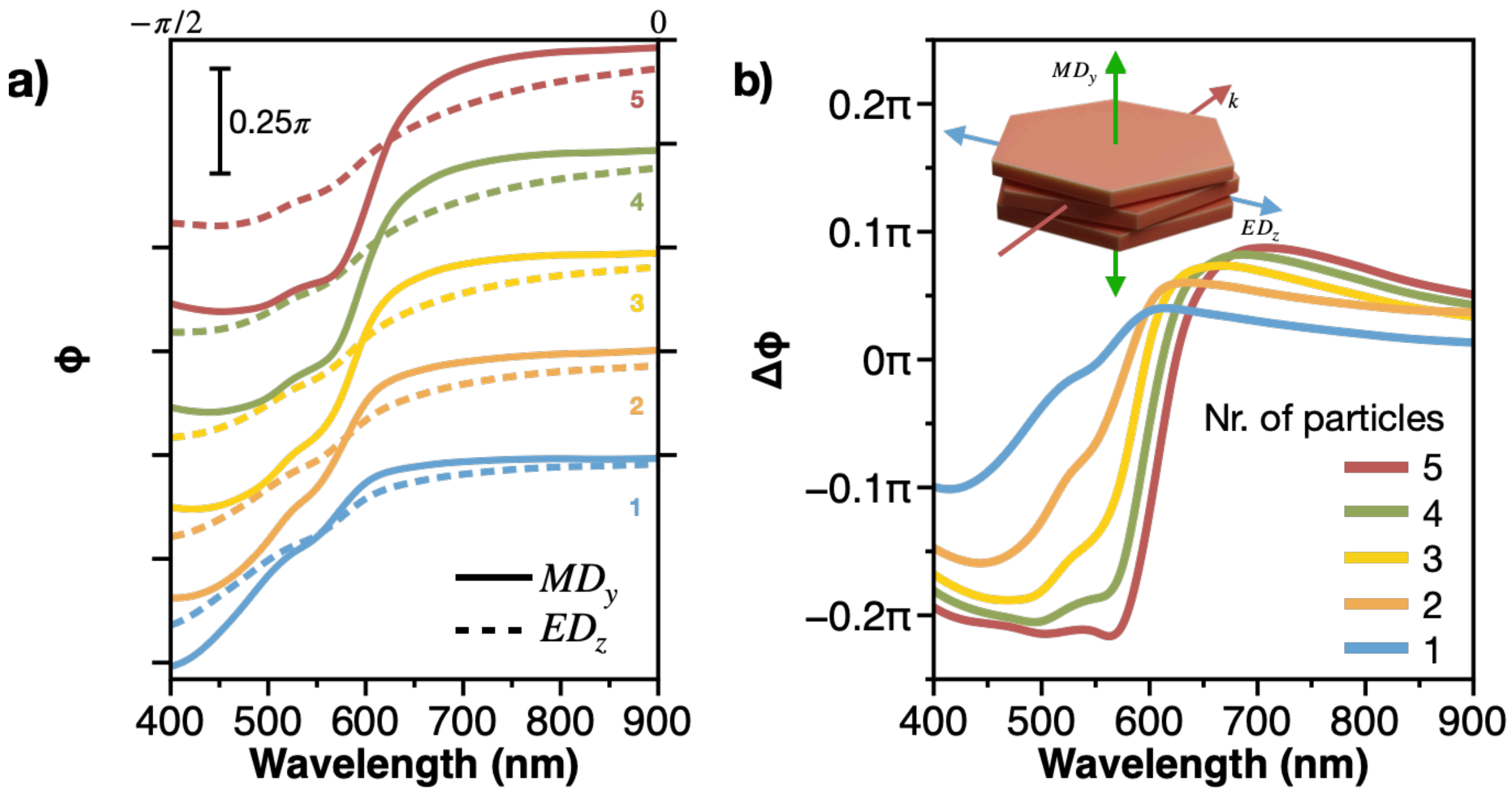

Figure S10. Phase of the magnetic (solid) and electric (dashed) field components for the in-plane excitation(the spectra are shifted by 0.25 for clarity). (a). Relative phase difference between the principal component of the magnetic and electric moments (MD_z - ED_y).

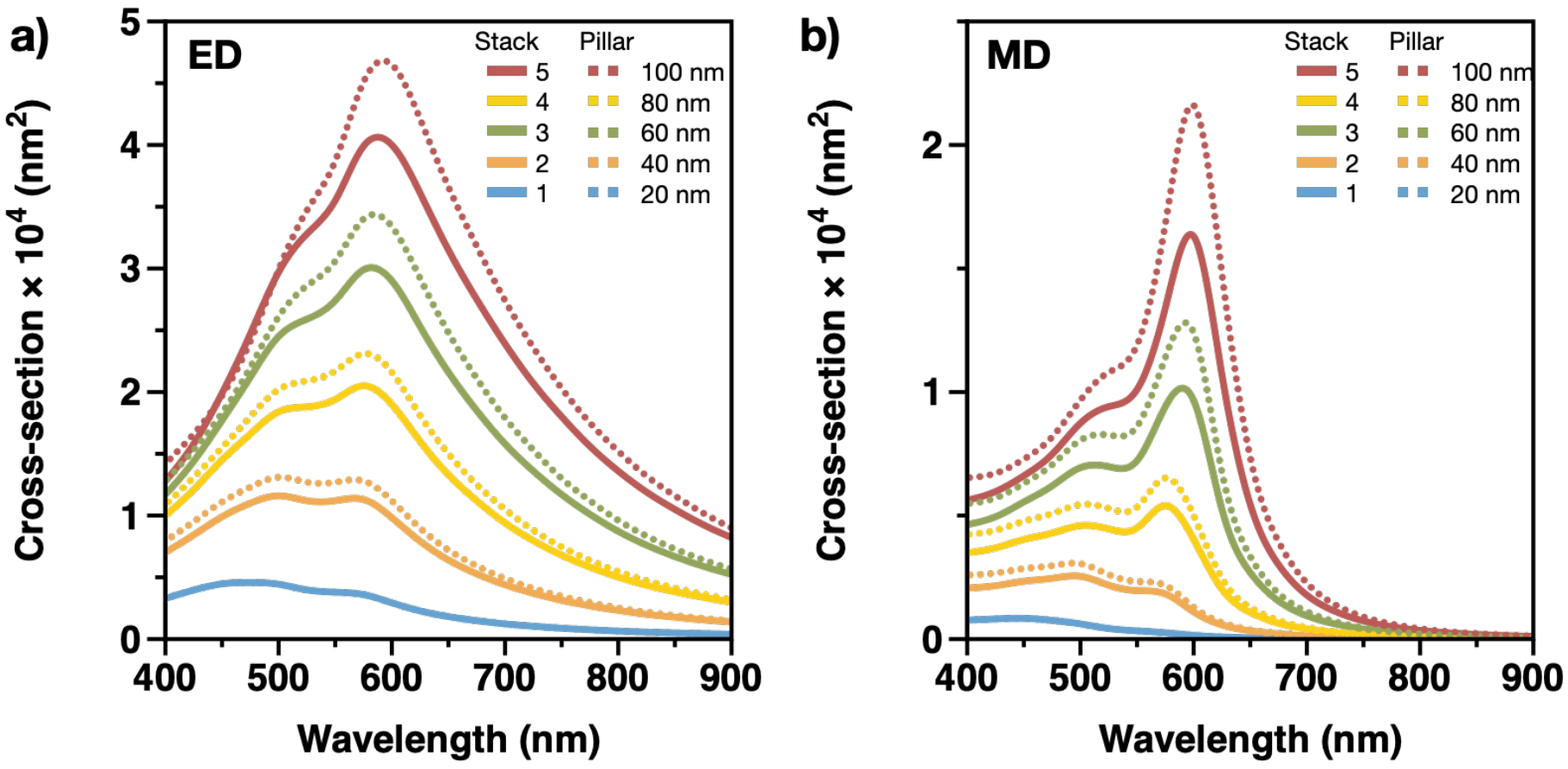

Figure S11. Comparison of the electric (a) and magnetic (b) dipole modes for the nanoplatelet stacks containing various numbers of 20 nm thick individual platelets with a nanopillar of equivalent height.

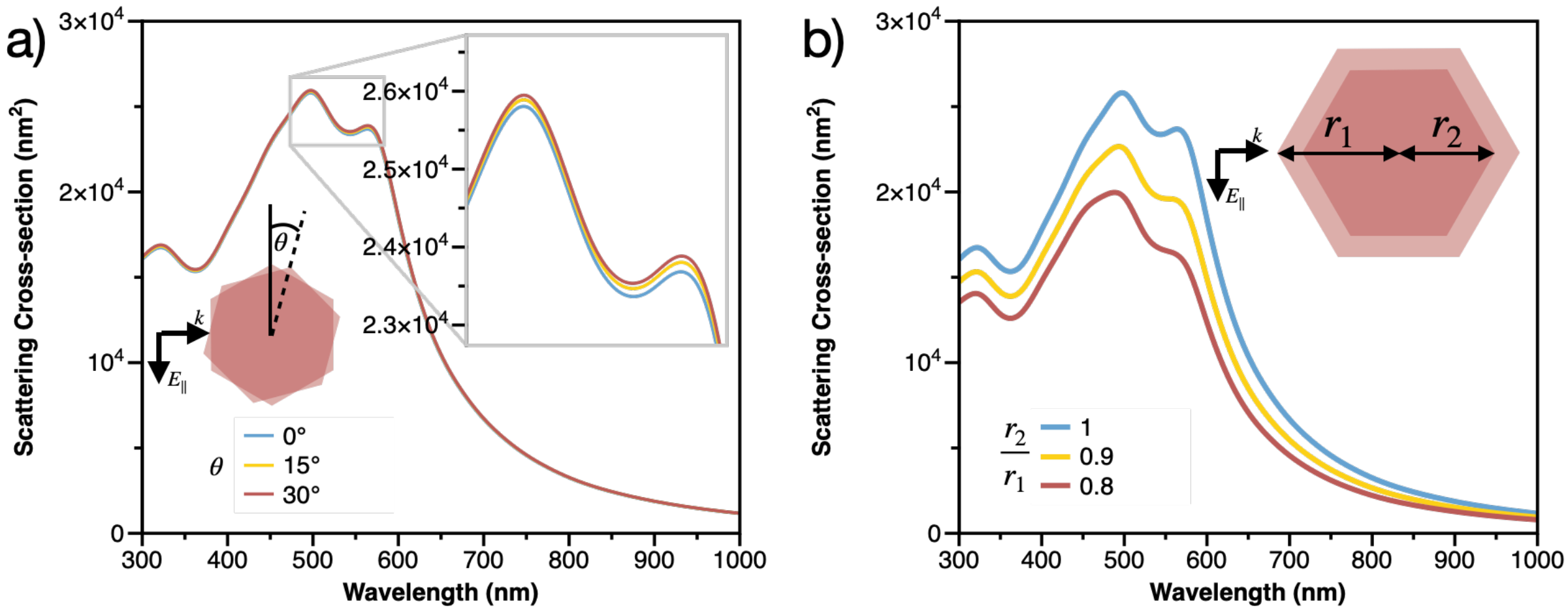

Figure S12. Simulated scattering spectra of a nanoplatelet dimer as a function of the angular displacement of the particles (a) and for a nanoplatelet dimer consisting of two particles of different sizes; r_1= 95 nm (b). Excitation in the plane of the dimer, particle gap: 2 nm.

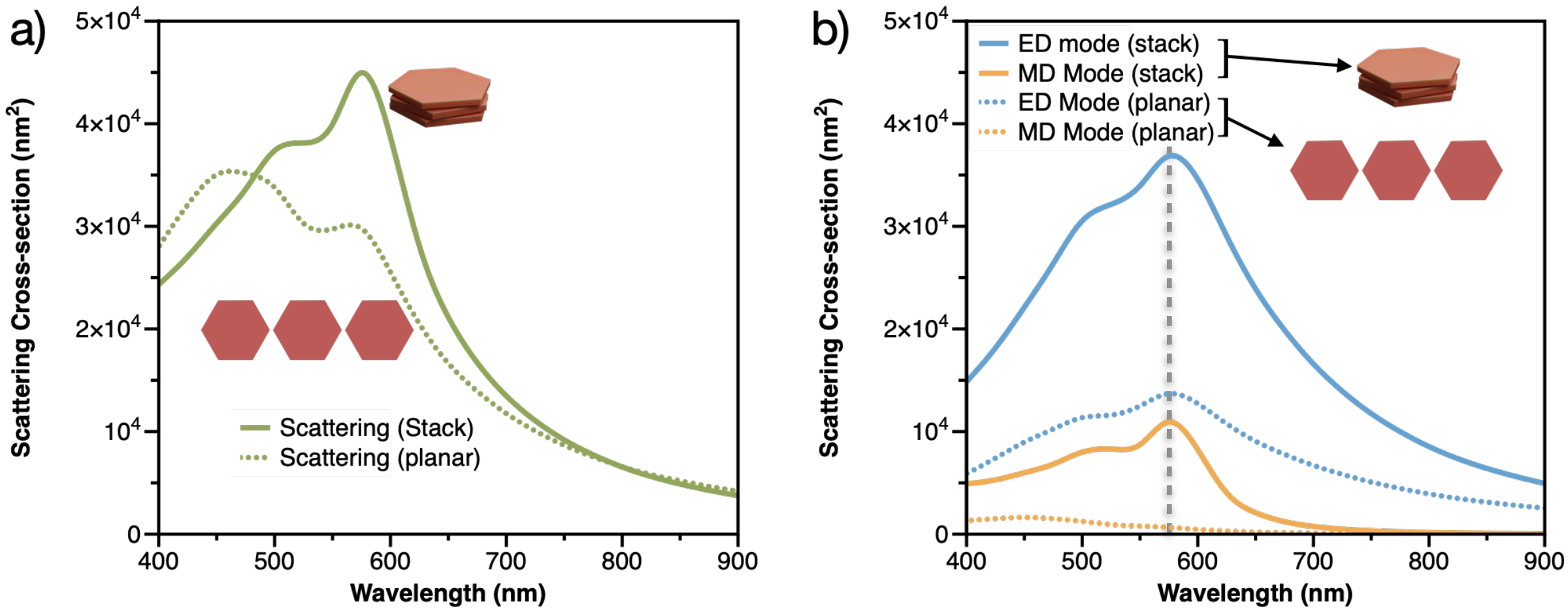

Figure S13. Comparison of the extinction spectra upon in-plane excitation of two platelet trimers stacked vertically and laterally (a) and the contribution of the electric and magnetic dipole modes to the scattering (b). The peak at 575 nm (associated with the collective magnetic dipole mode) is growing large for the vertical stack, while it is virtually absent for the planar arrangement.

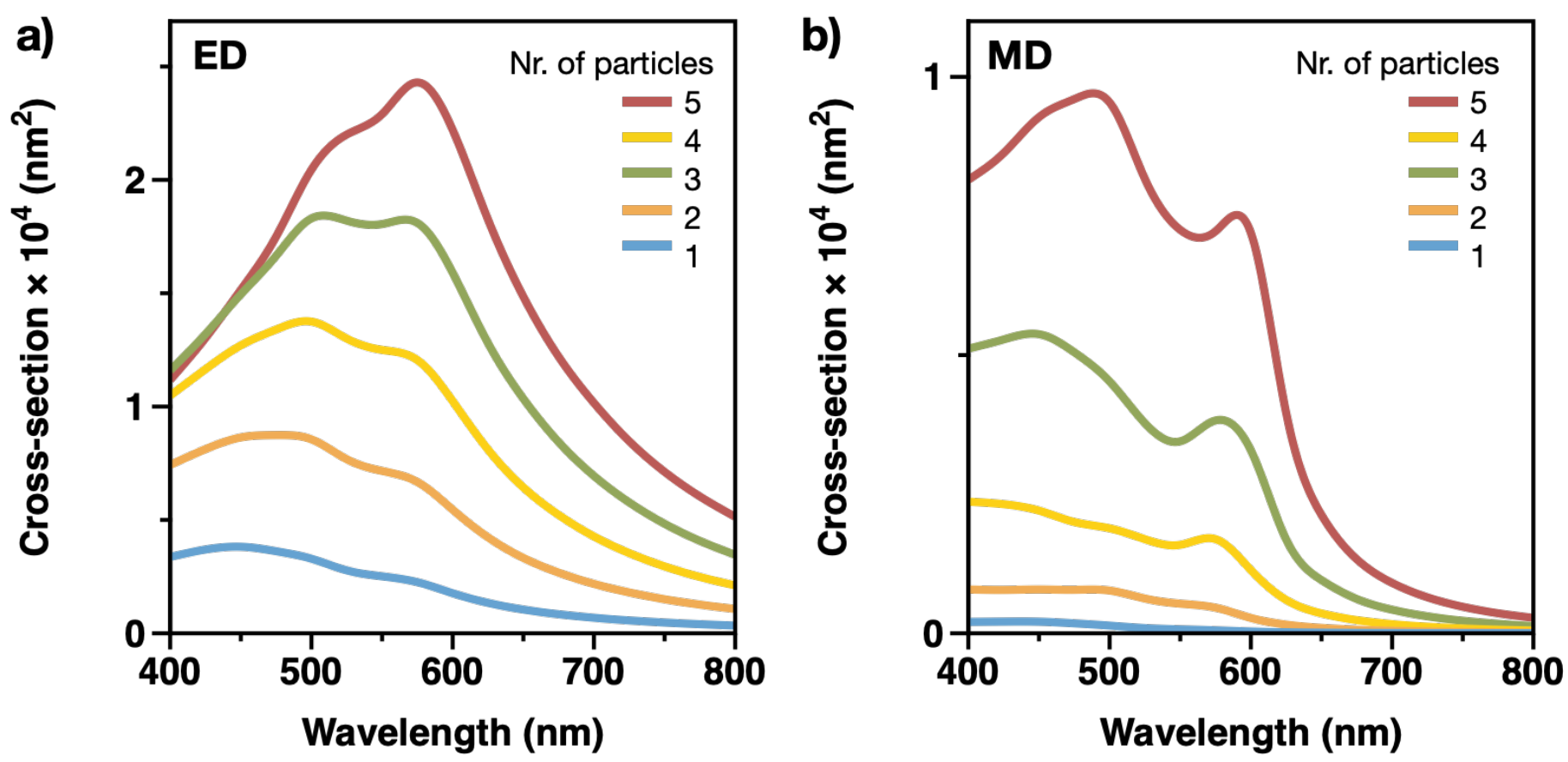

Figure S14. Evolution of the substrate supported stacks' electric (a) and magnetic (b) dipole modes' contribution to the scattering as a function of the number of particles forming the stack.

References

[1] M. R. Querry, *Optical Constants*, University Of Missouri-Kansas City, Kansas City, **1984**.
